# Integrating high-throughput analysis to create an atlas of replication origins in *Trypanosoma cruzi* in the context of genome structure and variability

**DOI:** 10.1128/mbio.00319-24

**Published:** 2024-03-05

**Authors:** Marcela de Oliveira Vitarelli, Thiago Andrade Franco, David da Silva Pires, Alex Ranieri Jerônimo Lima, Vincent Louis Viala, Amelie Johanna Kraus, Inácio de Loiola Meirelles Junqueira de Azevedo, Julia Pinheiro Chagas da Cunha, Maria Carolina Elias

**Affiliations:** 1 Cell Cycle Laboratory, Butantan Institute, Av. Vital Brazil, São Paulo, Brazil; 2 Center of Toxins, Immune Response and Cell Signaling (CeTICS), Butantan Institute, Av. Vital Brazil, São Paulo, Brazil; 3 Center of Scientific Division, Butantan Institute, Av. Vital Brazil, São Paulo, Brazil; 4 Biochemistry Laboratory, Butantan Institute, Av. Vital Brazil, São Paulo, Brazil; 5 Division of Experimental Parasitology, Faculty of Veterinary Medicine, Ludwig-Maximilians-Universität in Munich, Planegg-Martinsried, Germany; 6 Biomedical Center, Division of Physiological Chemistry, Faculty of Medicine, Ludwig-Maximilians-Universität in Munich, Planegg-Martinsried, Germany; 7 Applied Toxinology Laboratory, Butantan Institute, Av. Vital Brazil, São Paulo, Brazil; University of Geneva, Geneva, Switzerland

**Keywords:** replication origins, prereplication complex, *Trypanosoma cruzi*, DNA replication

## Abstract

*Trypanosoma cruzi* is the etiologic agent of the most prevalent human parasitic disease in Latin America, Chagas disease. Its genome is rich in multigenic families that code for virulent antigens and are present in the rapidly evolving genomic compartment named Disruptive. DNA replication is a meticulous biological process in which flaws can generate mutations and changes in chromosomal and gene copy numbers. Here, integrating high-throughput and single-molecule analyses, we were able to identify Predominant, Flexible, and Dormant Orc1Cdc6-dependent origins as well as Orc1Cdc6-independent origins. Orc1Cdc6-dependent origins were found in multigenic family loci, while independent origins were found in the Core compartment that contains conserved and hypothetical protein-coding genes, in addition to multigenic families. In addition, we found that Orc1Cdc6 density is related to the firing of origins and that Orc1Cdc6-binding sites within fired origins are depleted of a specific class of nucleosomes that we previously categorized as dynamic. Together, these data suggest that Orc1Cdc6-dependent origins may contribute to the rapid evolution of the Disruptive compartment and, therefore, to the success of *T. cruzi* infection and that the local epigenome landscape is also involved in this process.

**IMPORTANCE:**

*Trypanosoma cruzi*, responsible for Chagas disease, affects millions globally, particularly in Latin America. Lack of vaccine or treatment underscores the need for research. Parasite’s genome, with virulent antigen-coding multigenic families, resides in the rapidly evolving Disruptive compartment. Study sheds light on the parasite’s dynamic DNA replication, discussing the evolution of the Disruptive compartment. Therefore, the findings represent a significant stride in comprehending *T. cruzi*’s biology and the molecular bases that contribute to the success of infection caused by this parasite.

## INTRODUCTION

*Trypanosoma cruzi*, a kinetoplastida protozoan, is the etiologic agent of Chagas disease, the most prevalent human parasitic disease in Latin America. According to the Pan American Health Organization, it is estimated that approximately six million people in 21 countries were infected with *T. cruzi* (1). In addition, approximately 13% of the Latin American population is at risk of contracting *T. cruzi* infection due to vectorial and nonvectorial transmission. In addition, other geographic regions, such as North America, Europe, and Asia, are facing increased risk of Chagas disease due to the migration of infected people (2).

Many genetically different *T. cruzi* strains correlate with morphology, variance in parasitemia curves and mortality in murine models, therapeutic response to drugs, and tissue tropism. Before next-generation sequencing, it was shown that these strains, classified into six discrete typing units (DTU) TcI-TcVI (3), present variations in DNA content and in bands visualized by pulsed-field gel electrophoresis (4). Whole-genome sequencing of *T. cruzi* field isolates revealed that these differences could be the result of a complex aneuploidy pattern, as seen in TcII DTU (5).

The genome sequence of *T. cruzi* was published in 2005, and it turned out to be highly repetitive (repetitive sequences making up to 50% of the genome) (6, 7) due mainly to the presence of multigenic families generally located in large clusters (8). The most abundant in copy number are trans-sialidase (TS), mucin-associated surface protein (MASP), retrotransposon hot spot (RHS), mucins, GP63, and dispersed gene family 1 (DGF-1), which code for surface proteins that contribute to parasite-cell interaction and invasion and also to protection of the parasite from the host immune response. With advances in sequencing platforms, it became evident that the *T. cruzi* genome is compartmentalized into regions named Core and Disruptive (7), which are subject to different selective pressures. The first contains conserved and hypothetical genes displaying the same gene order (syntenic) as the *Trypanosoma brucei* and *Leishmania* genomes; the second presents higher GC content and is composed of rapidly evolving multigene families (9), such as TS, MASP, and mucins. GP63, DGF-1, and RHS are distributed in both compartments. The evidence of the rapid evolution of multigenic families was corroborated by a comparative analysis of 35 T. *cruzi* TcI strains, in which a higher density of single-nucleotide polymorphism (SNP) within multigenic family regions than in regions found in the Core compartment of the genome was found (10, 11).

Since genomic plasticity plays a key role in *T. cruzi* infection success, it is important to dissect the molecular bases that drive this plasticity. DNA replication is the process that warrants genomic maintenance; however, incomplete, erroneous, or premature DNA replication can generate mutations, chromosomal aneuploidy or polyploidy, and gene copy number variation (12, 13). In eukaryotes, DNA replication starts at several hundred (in budding yeast) to tens of thousands (in humans) regions called origins that are licensed from late mitosis to the G1 phase of the cell cycle when the prereplication complex (ORC complex, Cdc6, and Cdt1) binds these regions, loading the MCM2–7 replicative helicase motor in the form of a catalytically inactive head-to-head dimer around DNA (14). Then, when cells reach S-phase, the replisome (molecular machinery that carries out DNA replication) is recruited to some of these origins that become activated. Thus, two replication forks, provided by the head-to-head configuration of the MCM dimer, emerge from origins going in opposite directions until meeting another fork or the end of the chromosome (15, 16). According to their usage, origins are classified as (i) constitutive, which is activated in all cells of a population; (ii) flexible, whose activation is random; and (iii) dormant, which is passively replicated and only activated in the case of DNA damage or replicative stress (17), when the DNA replication fork is delayed or stalled (e.g., by the presence of secondary structures, repeats, z-DNA) (18). Except for *Saccharomyces cerevisiae*, eukaryotic origins do not present consensus DNA elements. Instead, their location is influenced by DNA structure, topology, and chromatin environment (19, 20). Although we can assume that because the entire genome must be duplicated, the location of origins is irrelevant, their position is crucial to coordinate DNA replication with other processes that use DNA as a template, such as transcription and DNA repair. Indeed, in metazoans, origins localize at intergenic and promoter regions (21, 22), suggesting the importance of origin location for the maintenance of genomic stability. Moreover, transcription has a direct role in the location of origins, inhibiting MCM loading or repositioning loaded MCM on chromosomes (21, 23, 24).

The genome organization of *T. cruzi*, which is extended to other kinetoplastids, is quite peculiar since every gene is transcribed as part of a polycistronic transcription unit (PTU) that can cover hundreds of genes. Moreover, these PTUs do not possess a clear promoter sequence using bidirectional start sites. Regions located between PTUs are classified as divergent strand switch regions (dSSRs) and convergent strand switch regions (cSSRs), which are the beginning and termination of PTUs, respectively (25, 26). Therefore, recent studies have begun to explore the location of replication origins in kinetoplastids. In *Trypanosoma brucei*, origins were found (by marked frequency analysis-seq) at SSRs (27) and were found at the same regions in two different strains (28), suggesting a relatively rigid usage of origins. This conservation could be due to the maintenance of parasite genome organization since origins located within transcription units could cause clashes between transcription and replication machinery. The same methodology used to detect origins in *T. brucei* was used in two species of *Leishmania* (*L. major* and *L. mexicana*). Only one origin was identified per chromosome, similar to *T. brucei*, within SSRs (29). However, through the use of a highly sensitive methodology (sequencing isolated small nascent DNA strands), 5,100 DNA origins were predicted across the *L. major* genome with no preferential localization at SSRs (30). This number of origins represents one origin for every two genes, and it was proposed that this methodology could detect origin usage across the cellular population, implying that the number of origins fired in each cell is much lower (31). In *T. cruzi*, we have detected origins at SSRs, but most of them within coding DNA sequences (32), suggesting that, in *T. cruzi*, origin location may have evolved to contribute to genetic variability (due to the clashes between transcription and replication machinery that generates DNA damage that might be repaired) or to control gene expression through inhibition of transcription by the prereplication complex in that region.

To gain a better understanding of *T. cruzi* replication dynamics, we combined two approaches based on high-throughput and single-cell analysis to map origins in the *T. cruzi* genome. By integrating the genome location of the prereplication complex component Orc1Cdc6 and the detection of fired origins, we propose an atlas of the location of Predominant, Flexible, and Dormant origins and Orc1Cdc6-free origins. Moreover, we found that the Orc1Cdc6 distribution changes between Predominant and Flexible origins as well as their epigenetic context.

## MATERIALS AND METHODS

### Cell culture

Epimastigotes from *T. cruzi* CL Brener wild type and Cas9 constitutively expressing Cas9 protein and T7 RNA polymerase promoter (33) strains were cultivated in liver infusion tryptose medium supplemented with 10% fetal bovine serum (Vitrocell) and 0.5% hemin at 28°C at a density of $5\times 10^{6}$ parasites/mL. The Cas9 strain was also maintained in G418 (100 μg/mL).

### CRISPR-Cas9 transfection

CRISPR-Cas9 was used for 3xTy1 tag addition in the N-terminal region of the Orc1Cdc6 (TcCLB.511159.20) protein. Double-stranded single guide RNA (sgRNA) amplification was performed *in vitro* by a long prime PCR (34) containing the T7 promoter, a 20-bp target sequence designed using the Eukaryotic Pathogen CRISPR gRNA Design Tool (35), and the sgRNA scaffold sequence (36). The following set of primers was used for sgRNA amplification, forward: 5′ GAA ATT AAT ACG ACT CAC TAT GGA tggcctctttctactcacag TTT TAG AGC TAG AAA TAG C 3′, and reverse: 5′ AAA AGC ACC GAC TCG GTG CCA CTT TTT CAA GTT GAT AAC GGA CTA GCC TTA TTT TAA CTT GCT ATT TCT AGC TCT AAA AC 3′, with the 20-nucleotide sgRNA target sequence represented in lowercase letters. Donor DNA was amplified by PCR using primers containing 30 bp homology in the target region of the genome and the pPOTv6-puro-puro-mNG:Ty1 vector (37). The following set of primers was used for donor DNA amplification, forward: 5′ TTG TCT GAA ATG GCA TTC TTA TAT TAC ATT GTA TAA TGC AGA CCT GCT GC 3′, and reverse: 5′ TTC ACC AAA AAC CAA AAT GAC TCG TGA ATT AAG CTT ATC CAA GGG ATC TTG ATT G 3′. Transfection and cloning were performed as described by Rosón et al. (38). Primers forward: 5′ GGA CTA TCG AAA TTC TTT TGA GCG TTA CTC ACG TTC AA 3′, and reverse: 5′ TGC CCA AGA CGA GCT TTC CT 3′, annealing in the 5′ UTR and in the Orc1Cdc6 gene, respectively, were used to confirm whether the tag was inserted correctly together with a Western blot assay using anti-Ty1 tag antibody (BB2 clone).

### Growth curves

Growth curves were generated in triplicate with a starting dilution of $1\times 10^{6}$ cells/mL. Cell density was monitored for 7 days in a Neubauer chamber.

### ChIP-seq

Three replicates of $3\times 10^{8}$ epimastigotes from *T. cruzi* CL Brener Cas9 and *T. cruzi* CL Brener Cas9 Orc1Cdc6-Ty1 were crosslinked with 0.5% formaldehyde for 20 min at RT, quenched with 2 M glycine, and washed with TDB buffer (5 mM KCl, 80 mM NaCl, 1 mM MgSO_4_, 20 mM Na_2_HPO_4_, 2 mM NaH_2_PO_4_, and 20 mM glucose pH 7.4). Cells were permeabilized with lysis buffer (1 mM potassium L-glutamate, 250 mM sucrose, 2.5 mM CaCl_2_, 1 mM PMSF) containing 0.1% Triton-X-100 for 15 min at RT, washed two times with lysis buffer, and resuspended in 1 mL lysis buffer containing 0.1% SDS. Upon permeabilization, each sample was sonicated in a Covaris S2 (5 W, 200 CPB, 10% DF) for 10 min at 4°C followed by centrifugation at 10,000 × *g* for 10 min at 4°C. Then, 300 mM NaCl was added to the supernatant, and an input sample was collected and stored at −20°C. The remaining sample was submitted to chromatin immunoprecipitation (ChIP) using 50 μL of Dynabeads protein G (Thermo Fisher) coupled with 2 μg of anti-Ty1 tag antibody (BB2 clone) (Invitrogen) for 16 h at 4°C with slow rotation. The beads were then washed eight times with cold RIPA buffer (50 mM HEPES-KOH pH 7.5, 500 mM LiCl, 1 mM EDTA, 1% NP-40, 0.7% Na-deoxycholate), one time with TE buffer containing 50 mM NaCl and eluted in elution buffer (10 mM EDTA, 50 mM Tris-HCl pH 8.0, 1% SDS) for 30 min at 65°C, followed by the addition of 300 mM NaCl. ChIP and input samples were incubated for 9 h at 65°C for crosslink reversal and treated with RNase A (0.2 mg/mL) for 2 h at 37°C and then with proteinase K (0.2 mg/mL) for 2 h at 55°C, followed by DNA purification using a PCR Clean-Up Gel Extraction Kit (Macherey-Nagel) and NTB buffer (Macherey-Nagel) for samples containing SDS. The final ChIP and input DNA samples were prepared using the Illumina TruSeq ChIP Library Preparation Kit and sequenced in an Illumina HiSeq 1500 (paired-end, 75 bp).

### ChIP-seq analysis

First, sequenced reads were submitted to adaptor trimming and quality filtering with Trimmomatic v.0.39 (LEADING:5 TRAILING:5 SLIDINGWINDOW:4:15 MINLEN:15) (39). To exclude the possibility of contaminants in the replicates, the first thousand reads of each mate of paired-end sample (*R*1 and *R*2) were aligned against the nonredundant (nr) NCBI database to check for exogenous contaminants using BLASTn (40), and a maximum of only 3.6% of total reads mapped in a different genome. Then, Trimmomatic-filtered reads were mapped against *T. cruzi* CL Brener and *T. cruzi* CL Brener Esmeraldo-like v.32, available at TriTrypDB (41, 42), with HISAT2 v.5.4.0 (- -score-min L,0.6,–0.6 - -no-spliced-alignment) (43). Genome coverage graphs of mapped reads were generated by converting BAM to BED and then to BIGWIG files. The search for ChIP-seq enriched regions was performed using HOMER v.4.11 (44), and the effective genome size generated by faCount (https://anaconda.org/bioconda/ucsc-facount), with the following parameters: (i) makeTagDirectory [-sspe -mapq 10 -tbp 1 (-unique|-keepAll)], (ii) findPeaks [-style (histone|factor) (to find the best output) -F 2 -fdr 0.05 -size 50 -minDist 150 -gsize 25827529], and (iii) mergePeaks (-d 340 -matrix -gsize 25827529). As cited above, the mergePeaks tool performs the concatenation of the three replicates. Control significant peaks were subtracted from those obtained for Orc1Cdc6-Ty1 to eliminate nonspecific binding to Ty1 antibody during ChIP.

### D-NAscent

A total of $1\times 10^{8}$ epimastigotes from *T. cruzi* CL Brener wild type were incubated with 300 μM BrdU for 30 min, and samples were washed with PBS and treated with proteinase K (50 μg/mL) and 0.5% SDS for 3 h at 50°C. High molecular weight DNA was extracted using the phenol:chloroform method (45). Sodium acetate (300 μM) was added to each sample prior to DNA precipitation with ethanol. Control and BrdU-incorporated DNA samples were treated with Short Read Eliminator (Circulomics), and nanopore sequencing libraries were constructed using the SQK-LSK 109 Oxford Nanopore Technologies kit. Sequencing was performed with 5 μg of DNA per run in an Oxford Nanopore MinION sequencer (FLO-MIN 106D v.R9).

### D-NAscent analysis

MinION reads were basecalled using Guppy v.4.2.2 (- -qscore filtering, - -trim_strategy dna) and mapped against the *T. cruzi* CL Brener Esmeraldo-like v.32 genome with Minimap2 v.2.17 (46) using default parameters. BrdU detection was performed using D-NAscent v.2.0.2 (47, 48) with the following parameters: (i) detect (- -quality 20 - -length 1000) and (ii) forkSense. D-NAscent software matches the electrical signal of BrdU with thymidine position and then determines the probability of a base being, in fact, BrdU. Then, the software estimates the probability of the fork direction based on the BrdU decay over the read. We first used the standard score (50%), determined for yeast, to determine the fork direction. However, the analysis of the first 100 reads using a graphical visualization tool (Integrative Genome Viewer) in order to check the fork direction showed 90% of false positives. Therefore, we had to change the score until we got a reduction of false positives to 22% (the minimum we got). Then, filtered reads with a fork direction probability equal to or higher than 70% were submitted to *ad hoc* scripts to determine replication regions, considering only replication forks with divergent directions within the same read. The interval between the start and end sites of the replication forks was considered the site of origin of replication. Since the origin size varied with the distance between replication forks, the center of each start site was determined, and a ±3-kb window was added to each coordinate, standardizing a 6-kb size for the origins of replication. Origin clustering was performed with the bedtools cluster function.

### The composition of the DNA replication origins atlas

The genomic coordinates of the database of D-NAscent, marked frequency analysis coupled with deep sequencing (MFA-seq), and Orc1Cdc6 ChIP-seq origins, as well as the intersect and subtract methods of bedtools v2.2.30.0 (49), enabled the development of four origin databases: Predominant, Flexible, Dormant, and Orc1Cdc6 free (for more information, see Fig. S1 to S4).

### Enrichment analysis of DNA replication origins

The repeated coordinates of the origins’ database were initially removed using the Linux sort command with the option -u for unique results. Then, an *ad hoc* script was created that compares the genomic coordinates of the replication origins with the genomic coordinates of the GFF file from the *T. cruzi* Esmeraldo-like v.32 genome (50) to determine where the replication origins are located. When the genomic coordinates of the origins intersect with a specific section of the genome, both coordinates are collected in a final file. The number of genomic coordinates of origins found in each given region of the genome is then determined. Finally, this origin distribution is compared to the expected standard frequency (considering the number of base pairs), which was determined based on its standard distribution in the genome.

### Origins distance estimation

An *ad hoc* script was developed to obtain the central point of genomic coordinates. In successive terms, we can say that this program adds the start and end coordinates of the origins, dividing the quotient by 2. The final quotient is defined as the start and end coordinates of DNA replication origins. Then, the subcommand closest, from bedtools v.2.30.0 (49), was used to identify the convergence and calculate the distance between the center point of the genomic coordinates of the origins.

### Heatmaps and hierarchical clustering

To determine the colocalization of the replication origins, Predominant and Flexible, with the Orc1Cdc6 ChIP-seq peaks, or the colocalization of Orc1Cdc6 (overlapping with the Predominant, Flexible, or Dormant origins) with nucleosomes, the computeMatrix and plotHeatmap tools were used from deepTools v.3.3.2 (51).

### Genome compartment composition

The compartments of *T. cruzi* genome were already stablished in the literature (7). In the initial phase of our investigation, we initiated isolating the “gene” feature from the *T. cruzi* CL Brener Esmeraldo-like v.32 genome as accessible through the TriTrypDB database (41, 42). This data set encompasses comprehensive genomic coordinates (only CDSs) for all annotated genes within the genome. Subsequently, we embarked on the task of segregating gene sequences belonging to the multi-gene families, namely TS, MASP, mucin, RHS, GP63, and DGF-1. This process led to the creation of distinct GFF files for each individual gene within these families. Following this, we merged the GFF files corresponding to the TS, MASP, and mucin genes, thereby establishing a comprehensive data set that contained the genomic coordinates for these three protein families, collectively referred to as the “Disruptive” compartment (Table S1). Simultaneously, we generated another GFF file comprising all gene sequences, except for those associated with the aforementioned multigenic families (TS, MASP, mucin, RHS, GP63, and DGF-1). This specific data set was designated as the “Core” compartment (Table S2). Since, the paper from Berná et al. (7) found DGF-1 and RHS and GP63 in both Core and Disruptve compartments, we introduced a third compartment exclusively dedicated to these proteins. In this way, to create this third compartment, we combined the GFF files that held the genomic coordinates for the RHS, GP63, and DGF-1 genes, culminating in the formation of the “Both” compartment (Fig. S5; Table S3).

### Statistical analysis

#### Chi-square

The standard frequency of the distribution of genome compartments, features, and CDS genes was first calculated. Thus, we observed that the standard frequency of the genome compartment was 83.04% Core, 13.10% Disruptive, and 3.86% Both compartments. The features have the following distribution: 2.97% cSSR, 5.78% dSSR, 0.33% inter-PTU, 90.78% CDS, 0.02% rRNA, 0.10% snoRNA, and 0.02% tRNA. Finally, the genes show the following distribution: 0.78% ATP dependent, 0.06% C/D, 0.31% Cysteine, 6.30% DGF-1, 0.20% Elongation, 0.37% Flagellar, 1.49% GP63, 48.13% Hypothetical protein, 0.13% Histone, 3.56% MASP, 1.29% Mucin, 0.14% Receptor type, 3.06% Retrotransposon, 6.65% TS, 0.18% UDP, and 27.27% others. The chi-square goodness-of-fit test was then performed using an R script and the previous frequencies as a standard. Additionally, the standardized residuals and adjusted standardized residuals were analyzed. The cutoff point for the standardized residuals, the *P* value for the residuals, and the effect size (Cramer’s *V*) were calculated (52, 53).

#### Wilcoxon-Mann-Whitney

The Wilcoxon-Mann-Whitney nonparametric test was used to compare the distances between the replication origins discovered by the methods D-NAscent or MFA-seq and the peaks of Orc1Cdc6 (ChIP-seq) (54). A similar approach was applied to compare the distance between the Predominant and Flexible origins of replication and the Orc1Cdc6 peaks.

### Search for Orc1Cdc6-binding motifs

The investigation of sequences that could work as motif for Orc1Cdc6 interaction was conducted using the MEME-ChIP software. Briefly, data sets containing genomic coordinates of Orc1Cdc6 coordinates at replication origins in BED format were converted using the bedtools getfasta tool, with the input being the genome version 32 of *T. cruzi* CL Brener Esmeraldo like. Subsequently, the MEME-ChIP software (Yeast, YEASTRACT, min 6, max 15), version 5.5.5, was employed (https://doi.org/10.1093/bioinformatics/btr189).

## RESULTS

To determine DNA replication dynamics and their epigenomic context in the *T. cruzi* CL Brener strain, we integrated four different methodologies: (i) ChIP-seq of Orc1Cdc6 to detect regions where prereplication machinery is bound to the genome (presented here), which corresponds to licensed origins; (ii) marked frequency analysis coupled with deep sequencing (32) to detect origins that fire in a high number of cells in a population; (iii) D-NAscent (presented here) to detect fired origins; and (iv) MNase-seq to evaluate nucleosome position and occupancy (55). *T. cruzi* CL Brener is a hybrid strain with homologous chromosomes presenting different lengths and genetic content and separated into two haplotypes: Esmeraldo-like and non-Esmeraldo-like haplotypes, both deposited in databases. Combined strategies of sequencing and mapping obtained 41 virtual chromosomes that are not the ideal *T. cruzi* reference genome, considering that other genome data sets are better assembled for other strains. However, we decided to perform these analyses in CL Brener (Esmeraldo-like haplotype) to compare the data obtained here with the previous analysis of our group: MFA-seq (32) and genome nucleosome positioning (55).

### Genome-wide distribution of Orc1Cdc6

Orc1Cdc6 is an origin licensing component of the *T. cruzi* prereplication machinery (56, 57). Therefore, we added a 3xTy1 tag at the 5′ region of this gene by CRISPR-Cas9 methodology to perform ChIP-seq experiments and detect prereplication machinery binding sites. We performed structure analysis of TcOrc1Cdc6 by I-TASSER, which showed a lower number of predicted secondary structures and a higher number of amino acids with an elevated exposition index at the N-terminal region compared with the C-terminal region, meaning that the tag at the 5′ region would have a greater probability of being exposed. Therefore, the *T. cruzi* CL Brener Cas9 lineage was transfected with sgRNA containing a target sequence for the 5′ UTR of Orc1Cdc6 and with donor DNA that was amplified using the vector pPOTv6-puro-puro-mNG:Ty1, which contains the resistance gene for puromycin and the 3xTy1 tag as a template (Fig. S6A). Parasites were selected and cloned, and PCRs and Western blots were performed to confirm the insertion of the tag. Figure S6B shows PCR fragments corresponding to the amplification of regions shown in Fig. S6A, evidencing the insertion of a tag at the 5′ region of Orc1Cdc6. Figure S6C shows 55-kDa bands corresponding to Orc1Cdc6-tag (50 kDa from Orc1Cdc6 and 5 kDa from tag). In addition, we confirmed that the 5′ tag at the N-terminal region did not interfere with parasite growth (Fig. S6D).

ChIP-seq assays using anti-Ty1 in lineages containing Orc1Cdc6 fused or not (control) to the Ty1 tag were performed in triplicate. On average, 12 million reads per replicate were sequenced, and 98% were recovered after adaptor removal and quality filtering. Then, these reads were mapped against the *T. cruzi* CL Brener genome, and a 33.7% mapping rate was obtained, on average, against the assembled Esmeraldo-like genome. This percentage might be due to (i) the lack of a non-Esmeraldo-like genome in this analysis and (ii) the exclusion of nonassembled sequences since we obtained approximately 70% reads of mapping against the entire nonassembled CL Brener genome (Table S4). While most of the entire chromosomes were covered, only the extremities of chromosome 38 were mapped; this result was also obtained for the input sample, and this fact is due to a huge gap in the middle of this chromosome in the reference genome. We analyzed Orc1Cdc6-binding sites in the genome by searching for peaks using the software HOMER. It performs statistical analysis to find significant peaks. In this analysis, we provided experimental samples (ChIP assay with anti-Ty1 in a lineage containing Orc1Cdc6 fused to Ty1), control samples (ChIP assay with anti-Ty1 in a lineage with no Ty1), and input data from three biological replicates with different parameters: one that searches for “histone pattern”—that identifies broad regions—and one that searches for “factor pattern,” which aims to identify the precise location of DNA-protein contact (44). Using the parameter “factor” and considering uniquely mapped peaks that were detected in all replicates, no peak was detected for the control sample or for the experimental sample. On the other hand, using the parameter “histone” and considering uniquely mapped peaks, we obtained almost the same peaks comparing control (22 peaks) and experimental samples (25 peaks) (Table S5), suggesting that we only obtained background peaks performing this analysis. Since the *T. cruzi* genome is highly repetitive and since we had previously detected origins in multigenic families (32), we decided to search peaks including multimapped reads. Then, we obtained 851 peaks from Orc1Cdc6-Ty1 after the subtraction of the control peaks (two peaks) (Tables S5 and S6).

Predicted Orc1Cdc6-binding sites present, on average, 537 bp (extracted from genomic coordinates where peaks are present provided by HOMER) totaling 526 kb (1.6% of the Esmeraldo-like haplotype genome), which is similar to *T. brucei* data that showed that Orc1Cdc6 occupies 2.4% of the genome (27) (this discrepancy could be due to *T. cruzi* genome annotation). In humans, the ORC2-binding site has been shown to have an average size of 550 nt (58). The median distance between *T. cruzi* Orc1Cdc6 peaks was 8,215 kb. We then analyzed a possible correlation between the distance of the peaks and the size of the chromosomes and between the number of peaks and the size of the chromosomes. In a linear regression model, we obtained *R*2 values of 0.0945 and 0.506 (Fig. S7A and B), respectively, the latter suggesting that larger chromosomes tend to have more licensed origins, while this is not true for all chromosomes. In addition, we noted the presence of peaks in chromosome extremities, suggesting the presence of Orc1Cdc6 at telomeres, as shown in *T. brucei* (27) (Fig. S8).

**Fig 1:**
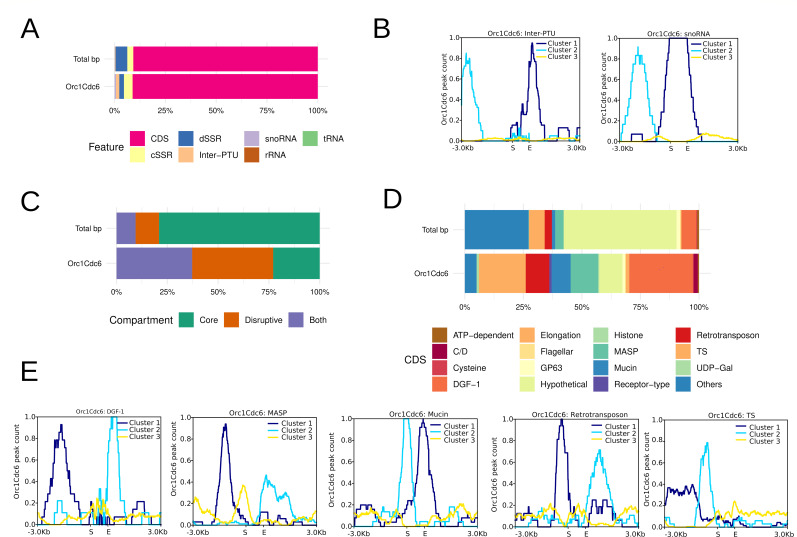
Genome-wide localization of Orc1Cdc6 peaks. (**A**) Distribution of Orc1Cdc6 peaks at different genome features. The chi-square goodness-of-fit test was used to perform statistical significance tests for the standard frequencies 2.97% cSSR, 5.78% dSSR, 0.33% inter-PTU, 90.78% CDS, 0.02% rRNA, 0.10% snoRNA, and 0.02% tRNA for given probabilities with simulated *P* value (based on 1,000 replicates) and post hoc test Cramer’s *V*. The values *P* = 0.000999, *X*^2^_(NA)_ = 97.823, and *V*-Cramer = 0.004499216 were obtained. (**B**) Hierarchical clusters for Orc1Cdc6 peak count mean concerning inter-PTU and snoRNA region considering a ±3-kb window. (**C**) Orc1Cdc6 peaks in genome compartments. Statistical significance tests were performed with the chi-square goodness-of-fit test using frequencies 83.04% Core, 13.10% Disruptive, and 3.86% Both as a reference. The values *P* = 2.2 × 10^−16^, *X*^2^_(2)_ = 2,378, and *V*-Cramer = 0.3940162 were obtained. (**D**) Distribution of Orc1Cdc6 peaks at CDS genes. The chi-square goodness-of-fit test was used to perform statistical significance tests for the standard frequencies 0.78% ATP dependent, 0.06% C/D, 0.31% Cysteine, 6.30% DGF-1, 0.20% Elongation, 0.37% Flagellar, 1.49% GP63, 48.13% Hypothetical, 0.13% Histone, 3.56% MASP, 1.29% Mucin, 0.14% Receptor type, 3.06% Retrotransposon, 6.65% TS, 0.18% UDP, and 27.27% others, for given probabilities with simulated *P* value (based on 1,000 replicates) and post hoc test Cramer’s *V*. The values *P* = 0.000999, *X*^2^_(NA)_= 1720.8 , and *V*-Cramer = 0.04098598 were obtained. (**E**) Summary plots of *k*-mean clusters for Orc1Cdc6 peaks concerning genes for multigenic family proteins (DGF-1, MASP, Mucin, Retrotransposon, and TS).

To map origins into the *T. cruzi* genome, we classified this genome into the following features: (i) loci of small RNAs (tRNA, snRNAs, snoRNAs, and rRNAs), (ii) polycistronic transcriptional units (PTUs) corresponding to protein-coding genes (that in the GFF version used here includes pseudogenes) (CDS), and (iii) sections between PTUs (dSSRs and cSSRs) or between PTUs and loci of small RNAs (inter-PTU) (6). Divergent strand switch regions and convergent strand switch regions serve as transcription start and stop regions, respectively (6, 59). Thus, Orc1Cdc6 peaks were classified according to *T. cruzi* genome features (Fig. 1A). We found Orc1Cdc6 peaks at different genome features, but statistical analysis utilizing the chi-square goodness-of-fit test considering the pattern distribution of features in the genome revealed enrichment of peaks in the inter-PTU and snoRNA loci (Fig. 1B). Therefore, we performed heatmap analysis to visualize the distribution of Orc1Cdc6 within these regions. In fact, we found that Orc1Cdc6 is in the center of snoRNA coordinates. Moreover, we also found Orc1Cdc6 in snoRNA neigborhood (in a window of 3 kb). On the other hand, Orc1Cdc6 was found within inter-PTU coordinates but displaced in the boundary of coordinates (Fig. 1B; Fig. S9). The neighborhood of snoRNA can be inter-PTU (where we, in fact, found Orc1Cdc6 peaks) or CDS. The boundary of inter-PTU can be CDS and small RNAs (in fact we found Orc1Cdc6 peaks in snoRNA). These results raised the possibility of the presence of Orc1Cdc6 in CDS.

In fact, we have previously identified fired origins at CDS, mainly at DGF-1 genes (32). Thus, we next investigated the presence of Orc1Cdc6 sites within genes. As described in the introduction, CDSs in the *T. cruzi* genome can be divided into two compartments, Core and Disruptive, based on the presence or absence of synteny among trypanosomatid lineages (7). Highly conserved genes with known functions and hypothetical conserved genes make up Core compartments (7). Genes for TS, mucin, and MASPs are among the Disruptive compartment (7, 60), and other genes from multigene families are scattered across the genome and fall into both categories, including retrotransposons, GP63, and DGF-1 (7). Therefore, we classified these genes into a Both compartment. The analysis of the distribution of Orc1Cdc6 peaks in the parasite genome compartments revealed an enrichment of the Orc1Cdc6 peaks in the Both and Disruptive compartments of the genome and a reduction in the Core compartment (Fig. 1C). When Orc1Cdc6 peaks into CDSs, their identity was examined (following annotation available in the GFF file from CL Brener version 32), and we observed that the great majority were identified in the area of multigene families (Fig. 1D). In this way, statistical analysis showed an enrichment of peaks in genes of multigenic family proteins (DGF-1, MASP, Mucin, Retrotransposon, and TS) and a decrease in C/D, Hypothetical, and others.

Analyses of hierarchical clusters followed by heatmap analysis improved visualization of the enrichment of Orc1Cdc6 peaks within multigene families and their vicinity (±3-kb window) (Fig. 1E; Fig. S9B). Orc1Cdc6 is present in the vicinity of TS, RHS, and MASP; it is upstream and downstream of the RHS, MASP, and TS coding regions, and there are large peaks upstream of TS. In addition, Orc1Cdc6 was found on top of the DGF-1, Mucin, and RHS coding regions and was also present in the vicinity of DGF-1 genes and downstream Mucin. These data demonstrate the presence of Orc1Cdc6 in the proximity of genes from multigene families. Interestingly, more than 85% of Orc1Cdc6-containing multigenic families are flanked by CDSs, evidencing the presence of Orc1Cdc6 inside CDSs. Also, Orc1Cdc6 were found within DGF-1, RHS, and Mucin, indicating the existence of the ORC complex in these locations and that replication initiation may be favored at these sites.

### Identification of Predominant, Flexible, and Dormant origins

In a previous work, we performed MFA-seq analysis in *T. cruzi* CL Brener (also known as Sort-seq in yeast) (61, 62), which has a detection threshold of 25% of the activity of the mapped origins (29) and thus detects only origins frequent in a population. In that work, we showed that some of these origins were located at cSSRs and dSSRs, but most of them were located at coding DNA sequences, preferentially at DGF-1 genes (32). In that article, we have raised the hypothesis that these origins within potentially transcribed regions could arise from transcription when the transcription machinery pushes the helicase MCM to new sites in addition to those licensed by ORC, as proposed in *Leishmania* (30).

**Fig 2:**
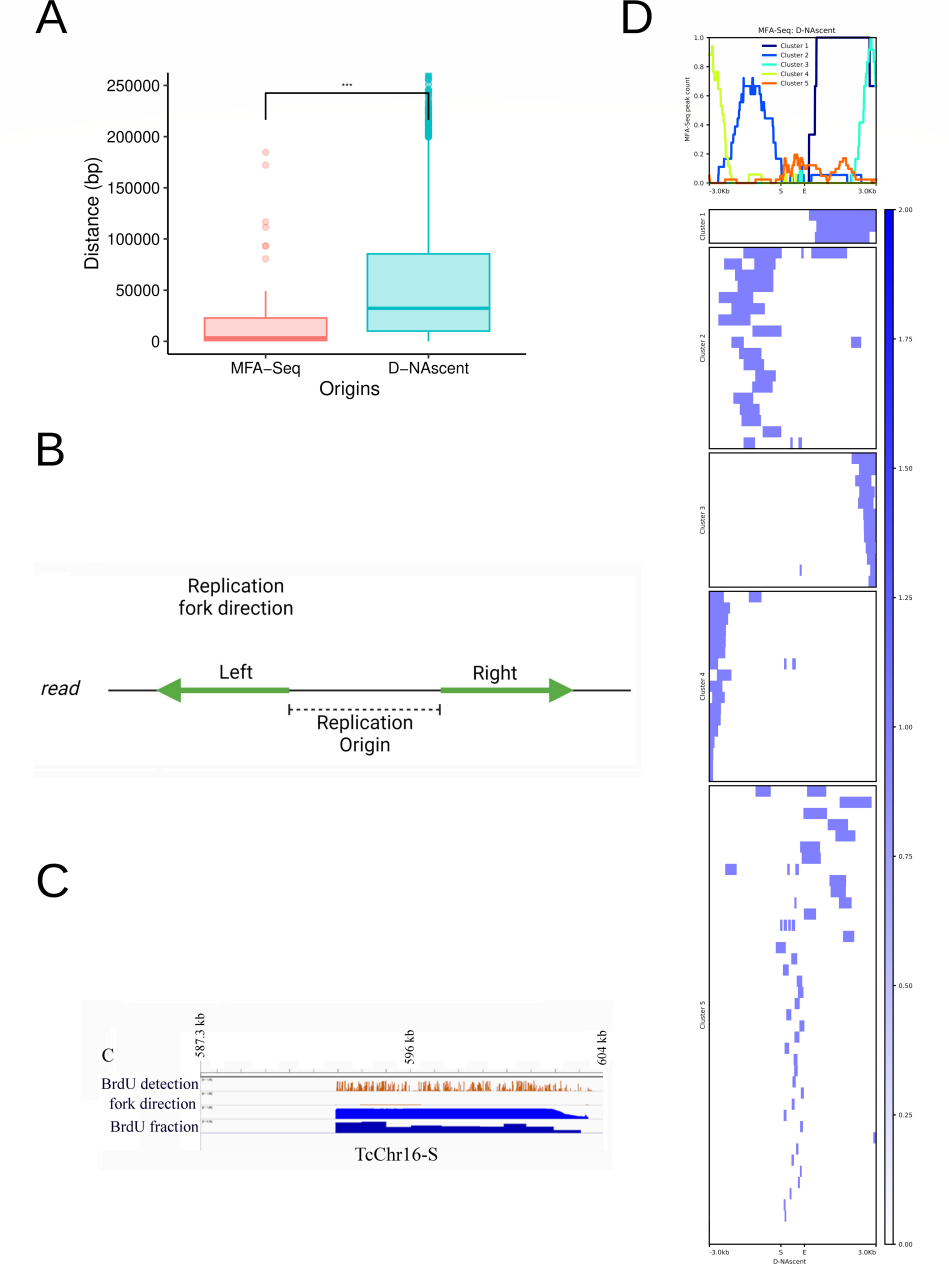
Characterization of D-NAscent-detected origins. (**A**) Distance distribution between the MFA-seq or D-NAscent origins and ChIP-seq-detected Orc1Cdc6 peaks. The center of each coordinate was used in this analysis. *** Statistical significance tests were performed with the Wilcoxon-Mann-Whitney test with *P* value = 0.0005348. (**B**) Scheme exemplifying the method used for the analyses of D-NAscent software products to pinpoint the origins of replication. The distance between two divergent sense replication forks in the same read was used to define origins of replication. Image created with BioRender.com. (**C**) Graphical representation of BrdU decay in a sequenced read. (**D**) Hierarchical clusters (five in total) and heatmap for MFA-seq origin peaks mean concerning D-NAscent origins considering a ±3-kb window.

Here, we analyzed which origins detected by MFA-seq were conventional ORC-defined origins and not origins fired (i) by MCM reaccommodation by transcription machinery (21) or (ii) by recombination-directed DNA replication initiation described in viruses, bacteria, archaea, and *Tetrahymena* [revised by Damasceno et al. (63)], especially because it has been proposed that recombination contributes to the evolution of multigenic families (64). Therefore, we searched for those MFA-seq-detected origins (Table S7) that matched Orc1Cdc6-binding sites (Table S8). The median distance between the MFA-seq-detected origin (center position) and the closest Orc1Cdc6 ChIP-seq peak (center position) was 3.4 kb (Fig. 2A). Analysis by hierarchical clustering showed that Orc1Cdc6 peaks are indeed constrained 3 kb around MFA-seq peaks, although we could see Orc1Cdc6 peaks up to 10 kb around MFA-seq-detected origins, while fewer Orc1Cdc6 peaks were detected between 10 and 20 kb from MFA-seq peaks (Fig. S10). From the 103 MFA-seq-detected origins, 29 overlapped with ChIP-seq peaks in at least 1 bp and 81 (78.6%) when we considered a window of ±3 kb from the center of the origin. We used the 81 MFA-seq-detected origins that matched with Orc1Cdc6 peaks (Table S8) to construct a data set named Predominant origins (see workflow at Fig. S1), meaning those origins containing Orc1Cdc6 detected in a high number of cells from a population.

As cited before, analysis of a population does not allow the detection of Flexible origins, which are activated in single or few cells. Although our previous results suggest that MFA-seq-detected origins would be enough to replicate the entire genome (32), the presence of origins within CDSs was associated with DNA replication fork collapse, DNA double-strand break formation, and chromosomal rearrangements (65). Thus, we analyzed if *T. cruzi* uses Flexible origins (not detected by MFA-seq) to preserve DNA maintenance. We also considered the possibility that Flexible origins might contribute to *T. cruzi* DNA replication dynamics even under nonstressed conditions. Therefore, we standardized the recently described D-NAscent methodology for *T. cruzi*. This method was developed to follow the DNA replication of individual molecules. The MinION nucleotide sequencing occurs due to the recognition of the electric profile of nucleotides moving through a pore. Using D-NAscent software, the recognition of the thymidine analog BrdU incorporated in replicating molecules was also detected (47). The decay of BrdU incorporation during the experiment allows the inference of the direction of the replication fork. Therefore, since two forks in opposite directions emerge from a replication origin, the replication origin is detected when divergent forks are found in the same molecule (Fig. 2B). Thus, high molecular weight DNA was extracted from asynchronous culture labeled or not with BrdU, extracted, and native genomic DNA (PCR free) was sequenced on a MinION (Oxford Nanopore Technologies). A total of 599,000 reads per sample were obtained on average (Table S9), and electrical signals were converted to nucleotides. Reads were then mapped against the *T. cruzi* CL Brener Esmeraldo-like genome, showing a mapping rate of approximately 83% (Table S9). In this work, we obtained reads with an average length of 6.2 kb and a median of 1.5 kb. The reads were then submitted to D-NAscent analysis, and Fig. 2C shows an example of a BrdU-positive read and inference of fork direction. We identified 4,287 origins (32.09% of BrdU-positive reads) ranging from 200 to 50,000 bp (Table S10). To limit that size, we took a ±3-kb window from the central point of the origin and compared it to the distance with the center of the closest Orc1Cdc6 ChIP-seq peak: 66.1 kb (average) and 32.3 kb (median) (Fig. 2A). We checked if some D-NAscent-detected origins matched with MFA-seq-detected origins by heatmap followed by hierarchical clustering in a window of 3 kb. Indeed, the majority of MFA-seq origins are detected by both methodologies when we consider a window of 3 kb (Fig. 2D). Then, we excluded from D-NAscent-detected origins those that were classified as Predominant (resulting in 4,253 origins) and performed an intersection analysis to find D-NAscent origins that did not match with MFA-seq origins but matched with Orc1Cdc6-binding sites. The 667 resulting origins were named Flexible, meaning conventional ORC-defined flexible origins (see workflow at Fig. S2; Table S11). Finally, Orc1Cdc6-binding sites that do not match with fired (MFA-seq or D-NAscent) origins were classified as Dormant (450 peaks—Table S12) (see workflow at Fig. S3). Additionally, from the 4,287 D-NAscent-detected origins (Table S10), 3,782 did not match with Orc1Cdc6 peaks; therefore, we called them Orc1Cdc6-free origins (Fig. S4; Table S13).

We then searched for sequences that could work as motif for Orc1Cdc6-DNA interaction using Orc1Cdc6-binding sites present in the three origins data set (Predominant, Flexible, and Dormant) (Fig. S11A). Then, we analyzed if the motifs found are some already described motif. While motifs found as Orc1Cdc6-binding sites from Predominant origins did not match with any known motif, we found one motif working as Orc1Cdc6-binding site from Flexible origins and three motifs working as Orc1Cdc6-binding sites from Dormant origins that match with motifs found in transcription factors (Fig. S11B). Interestingly, we got one motif, that is a motif for Azl1p transcription factor, present in both Flexible and Dormant origins.

### Genome-wide distribution of Predominant, Flexible, and Dormant origins

**Fig 3:**
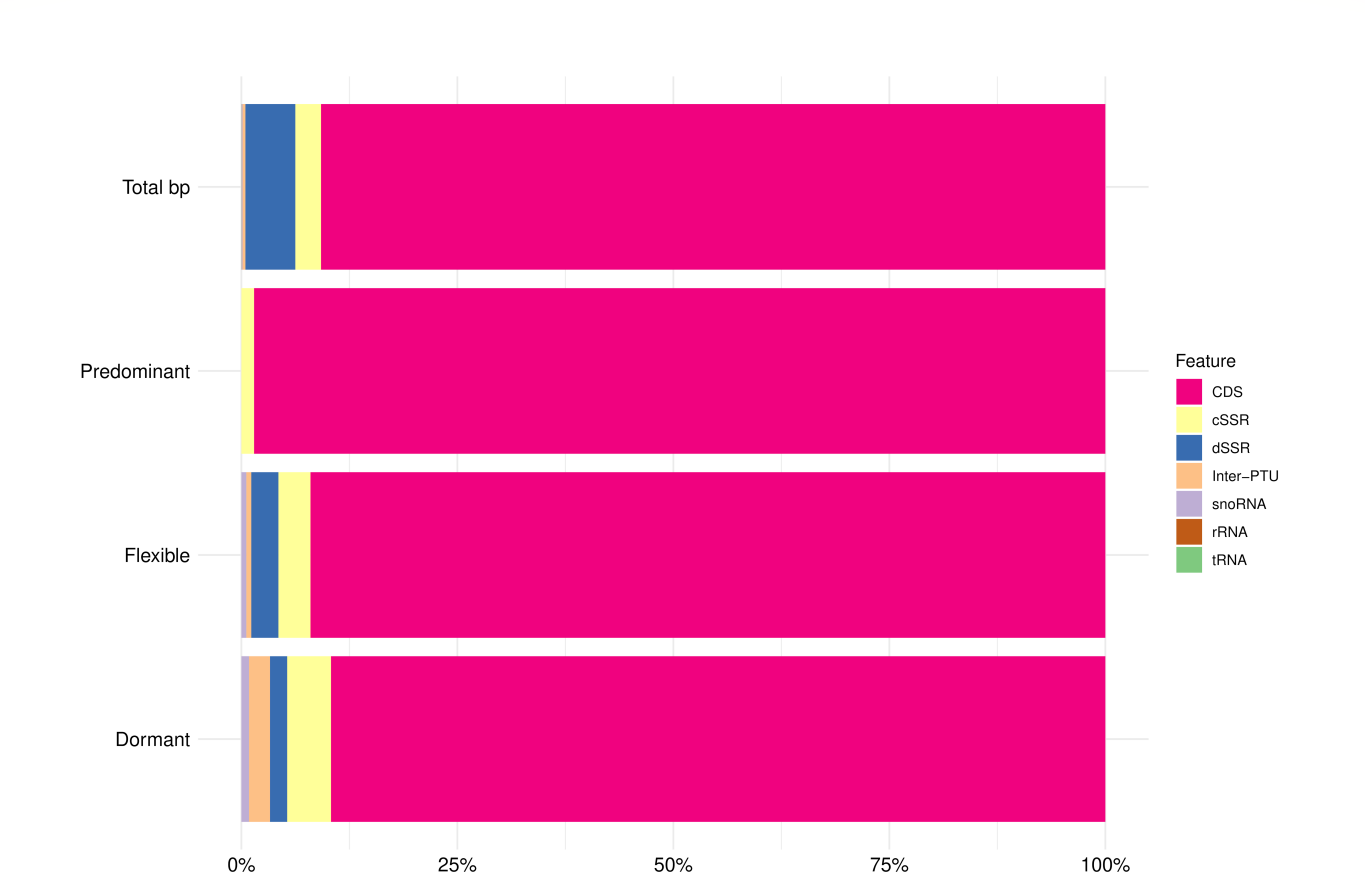
Distribution of Predominant, Flexible, and Dormant origins at different genomic features. The chi-square goodness-of-fit test was used to perform statistical significance tests for the standard frequencies 2.97% cSSR, 5.78% dSSR, 0.33% inter-PTU, 90.78% CDS, 0.02% rRNA, 0.10% snoRNA, and 0.02% tRNA for given probabilities with simulated *P* value (based on 1,000 replicates) and post hoc test Cramer’s *V*. The values *P* = 0.2278, ${X^{2}}_{\left(6\right)}=5.2118$, and *V*-Cramer = 0.003714641 to Predominant; *P* = 0.1429, ${X^{2}}_{\left(6\right)}=13.779$, and *V*-Cramer = 0.002662303 to Flexible; *P* = 0.000999, *X*^2^_(NA)_ = 107.32, and *V*-Cramer = 0.006530673 to Dormant were obtained.

We then analyzed the distribution of the Predominant, Flexible, and Dormant origins throughout the genome. To perform this analysis, we excluded the duplicated origins present in the Predominant, Flexible and Dormant data sets and used the coordinates of Orc1Cdc6 peaks present in these data sets (Fig. S1 to S3). We decided to use the coordinates of Orc1Cdc6 peaks instead of using origin coordinates since the length of Orc1Cdc6-binding sites is shorter than the origin length, thus presenting a more specific location in the genome. Interestingly, when we examined the distribution of Predominant replication origins, we noticed no significant enrichment of these origins in any genome feature. We found an enrichment of Flexible and Dormant origins in snoRNAs and an enrichment of Dormant origins in the inter-PTU (Fig. 3).

**Fig 4:**
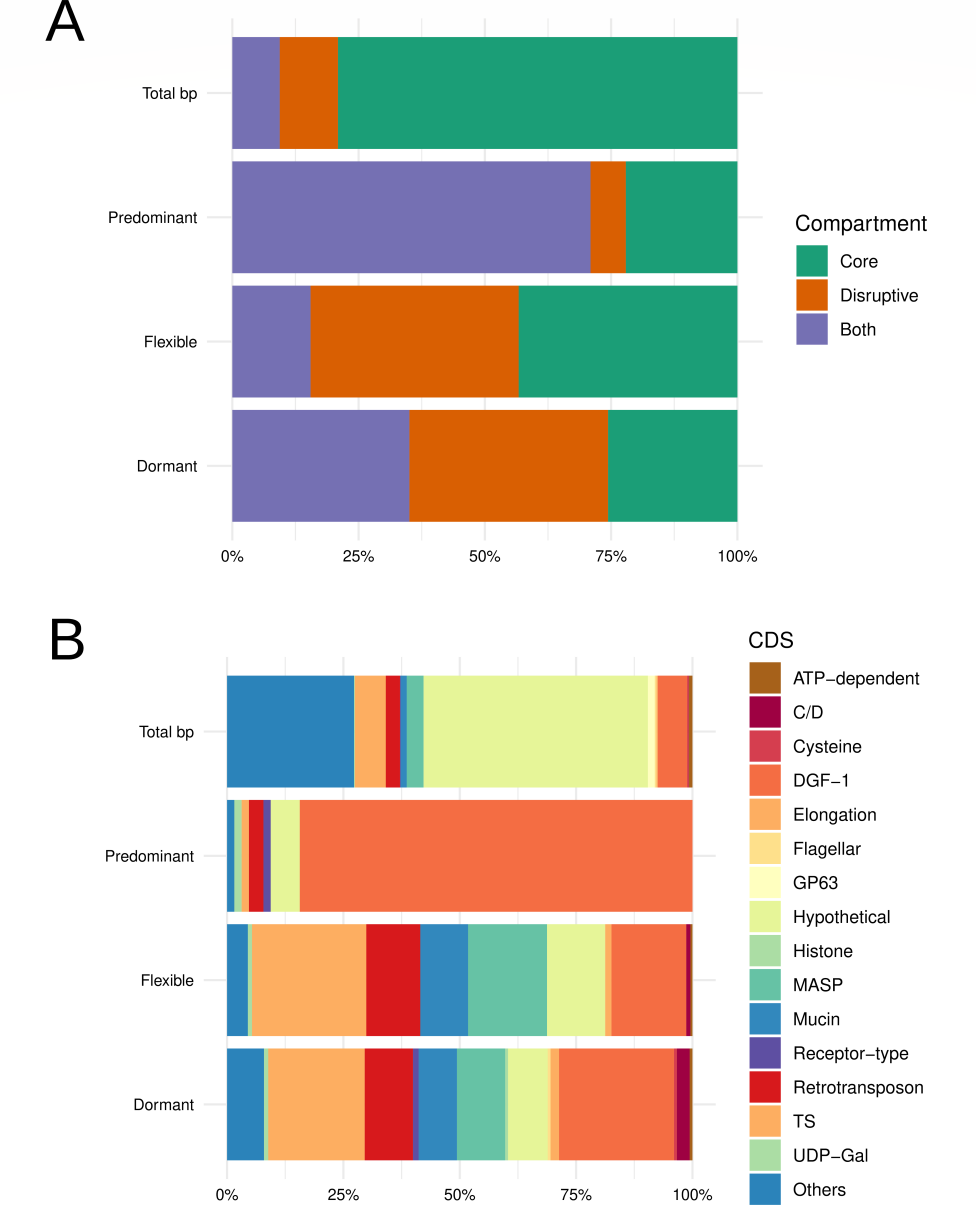
Distribution of Predominant, Flexible, and Dormant origins in genome compartments and CDS genes. (**A**) Distribution of Predominant, Flexible, and Dormant origins at genomic compartments. Statistical significance tests were performed with the chi-square goodness-of-fit test using the standard frequency 83.04% Core, 13.10% Disruptive, and 3.86% Both, and post hoc test Cramer’s *V*. The values *P* = 2.2 × 10^−16^, ${X^{2}}_{\left(2\right)}=1137.7$, and *V*-Cramer = 0.7463624 to Predominant; *P* = 2.2 × 10^−16^, ${X^{2}}_{\left(2\right)}=1304.7$, and *V*-Cramer = 0.2144572 to Flexible; *P* = 2.2 × 10^−16^, ${X^{2}}_{\left(2\right)}=1094.3$, and *V*-Cramer = 0.3665591 to Dormant were obtained. (**B**) Distribution of Predominant, Flexible, and Dormant origins at CDS genes. The chi-square goodness-of-fit test was used to perform statistical significance tests for the standard frequencies 0.78% ATP dependent, 0.06% C/D, 0.31% Cysteine, 6.30% DGF-1, 0.20% Elongation, 0.37% Flagellar, 1.49% GP63, 48.13% Hypothetical, 0.13% Histone, 3.56% MASP, 1.29% Mucin, 0.14% Receptor type, 3.06% Retrotransposon, 6.65% TS, 0.18% UDP, and 27.27% others, for given probabilities with simulated *P* value (based on 1,000 replicates) and post hoc test Cramer’s *V*. The values *P* = 0.000999, *X*^2^_(NA)_ = 681.91, and *V*-Cramer = 0.07997963 to Predominant; *P* = 0.000999, *X*^2^_(NA)_ = 597.2, and *V*-Cramer = 0.04000745 to Flexible; *P* = 0.000999, *X*^2^_(NA)_ = 1113.8, and *V*-Cramer = 0.04535975 to Dormant were obtained.

Concerning genome compartments, there is an enrichment of Predominant origins in multigenic families belonging to the Both compartment and an enrichment of Flexible and Dormant origins in the Both and Disruptive compartments (Fig. 4A). Statistical analysis showed an enrichment of Orc1Cdc6-free origins at the Core compartment and a decrease in the Both and Disruptive ones (Fig. S12). Then, we analyzed which genes these origins were located in, and we discovered, as expected, an enrichment of Predominant origins in DGF-1 genes, while we found a significant enrichment of Flexible and Dormant origins in multigenic family proteins (DGF-1, MASP, Mucin, Retrotransposon, and TS) and a down representation in hypothetical proteins and others that include all housekeeping genes crucial for organism operation (Fig. 4B). Then, we asked if Flexible and Dormant origins just reflect the general Orc1Cdc6 genome distribution. Therefore, we searched for regions where different classes of origins were enriched, considering the distribution of origins in the genome without breaking into the Predominant, Flexible, and Dormant (which means Orc1Cdc6 peaks) as the control group. The enrichment of origins in different regions found by statistical analysis is present in Tables 1 and 2. Since we saw enrichment of Flexible and Dormant origins in some regions, these data show that these origins do not follow the distribution of Orc1Cdc6 in the genome.

**TABLE 1:** Summary of enrichment of Orc1Cdc6 in *T. cruzi* CL Brener Esmeraldo-like genome features

Class of origin	Region where Orc1Cdc6 is enriched
Predominant	CDS
Flexible	CDS
	snoRNA
Dormant	CDS
	Inter-PTU
	snoRNA

**TABLE 2:** Summary of enrichment of Orc1Cdc6 in *T. cruzi* CL Brener Esmeraldo-like multigenic families

Class of origin	Genes where Orc1Cdc6 is enriched
Predominant	DGF-1
Flexible	Retrotransposon
	TS
	MASP
Dormant	Retrotransposon
	TS
	MASP

### Orc1Cdc6 density is different between Predominant and Flexible origins

**Fig 5:**
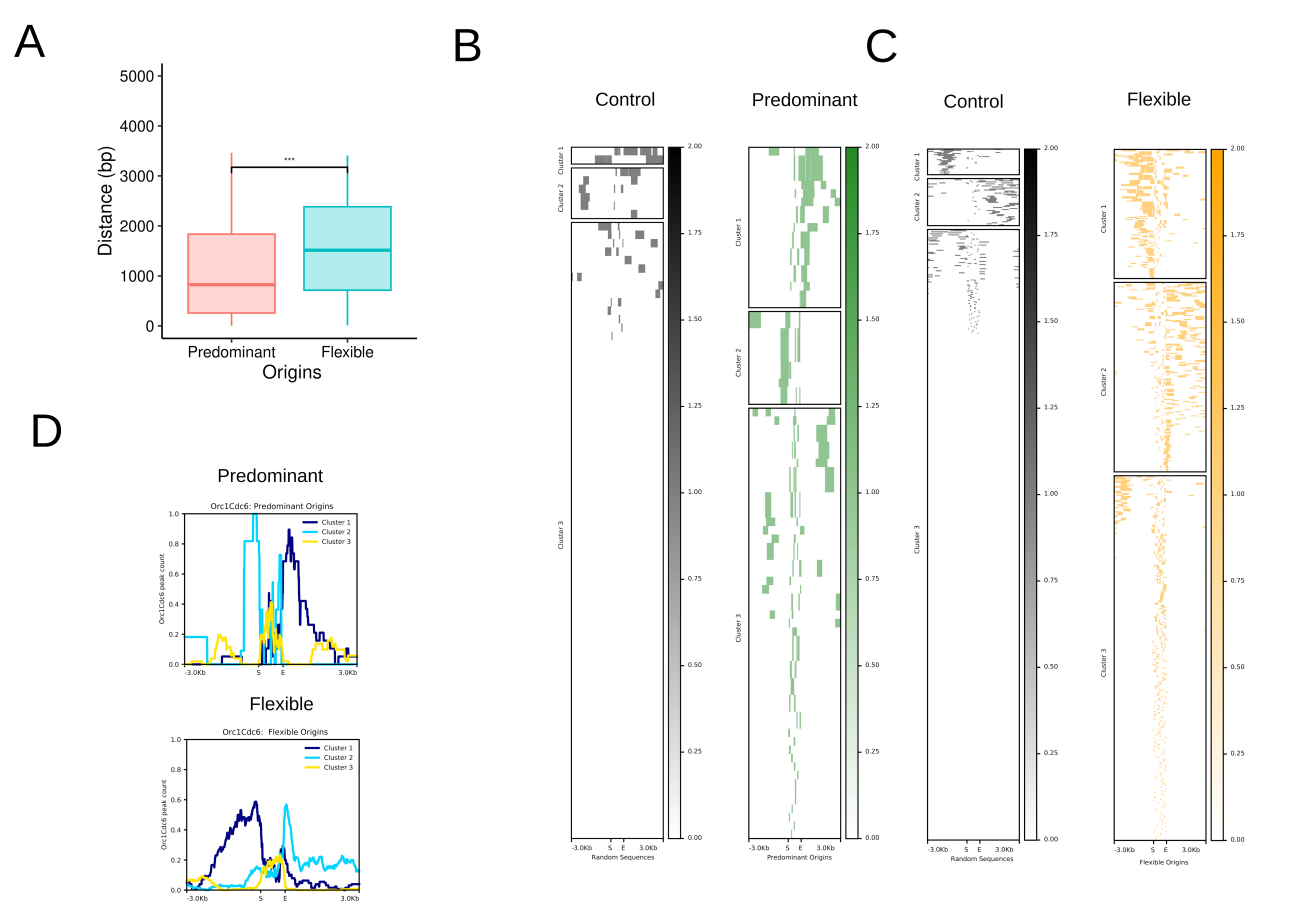
Orc1Cdc6 peaks at Predominant and Flexible origins. (**A**) Distribution of the distances between Predominant and Flexible origins to Orc1Cdc6-ChIP-seq peaks. The center of each coordinate was used in this analysis. Statistical significance tests were performed with the Wilcoxon-Mann-Whitney test with $P\;value=2.2\times 10^{-16}$. (**B**) Heatmaps for Orc1Cdc6 peak mean relative to Predominant origins (Predominant) or random sequences (Control) considering a ±3-kb window. Random sequences were selected at least three times. (**C**) Heatmaps for Orc1Cdc6 peak mean concerning Flexible origins (Flexible) or random sequences (Control) considering a ±3-kb window. Random sequences were selected at least three times. (**D**) Summary plots of the hierarchical clusters (*k*-means) for Orc1Cdc6 peaks concerning Predominant and Flexible origins considering a ±3-kb window.

A paradigm for the regulation of origin firing remains to be described but involves both MCM loading and activation (66). It has been suggested that early-firing origins in yeast rely on an average of three MCM double hexamers (67), showing that the more MCM loaded, the higher the probability of an origin being fired. In addition, accumulated evidence has indicated a correlation between ORC occupancy and origin activity, possibly by regulation of the stoichiometry of MCM loading in yeast [revised by Rhind (66)]. Since we have obtained Predominant and Flexible origins, meaning origins fired with more (Predominant) or less (Flexible) frequency, and we also obtained the presence of Orc1Cdc6 in these genomic loci, we then analyzed what would be the density of Orc1Cdc6 within these origins and in their neighborhood. To answer these questions, we first analyzed the distance between the center of the coordinates of Orc1Cdc6 peaks and the center of origins. We found that Orc1Cdc6 is closer to the center of the Predominant origin than the center of the Flexible origin (Fig. 5A). Then, we performed a heatmap analysis followed by hierarchical clustering searching the number of Orc1Cdc6 peaks within origins and in an additional ±3-kb window. As a control, we used randomly selected Esmeraldo-like sequences containing the same number and average size of origins (81 for Predominant and 667 for Flexible) (Fig. 5B through D). As expected, a very reduced number of random sequences colocalized with Orc1Cdc6-binding sites (see controls in Fig. 5B and C). Interestingly, while Predominant origins contain Orc1Cdc6 within origins and in their boundaries (<1 kb), Flexible origins contain Orc1Cdc6 spread throughout their neighborhood (Fig. 5D). These data suggest that Orc1Cdc6 density contributes to the frequency of origin firing.

### Correlation between origins and nucleosome positioning and occupancy

**Fig 6:**
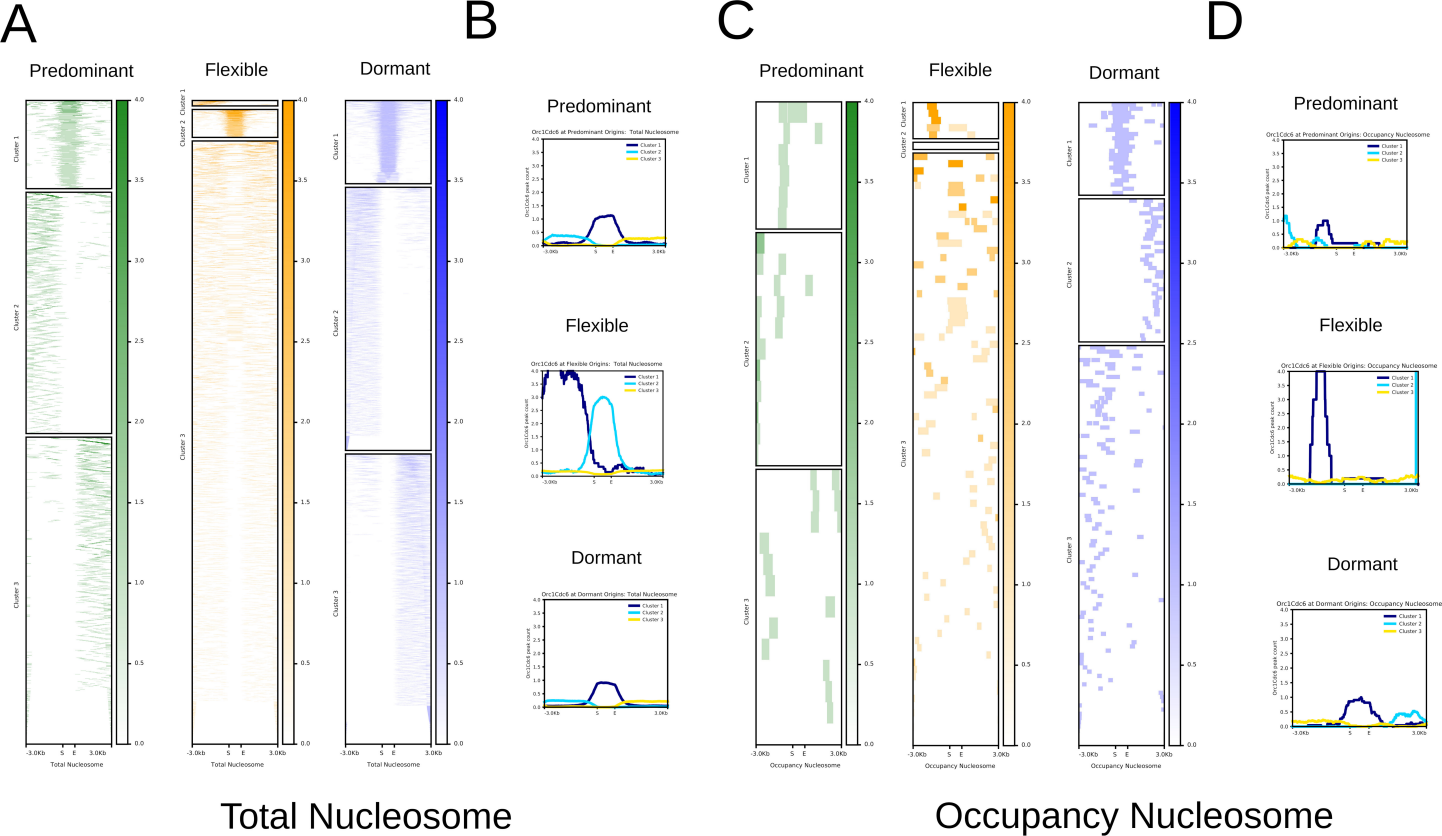
Co-localization of Orc1Cdc6 peaks mean at Predominant, Flexible, and Dormant origins in relation to total and occupancy nucleosomes. It was considered a ±3-kb window at all analyses. (**A**) Heatmaps relative to total nucleosomes. (**B**) Hierarchical clusters relative to total nucleosomes. (**C**) Heatmaps relative to occupancy nucleosomes. (**D**) Hierarchical clusters relative to occupancy nucleosomes.

Evidence has emerged pointing to the correlation between ORC loading and nucleosome location. The stability and precise positioning of nucleosomes affect replication control. Replication origins are mainly located within nucleosome-free regions since nucleosomes can reduce origin licensing by inhibiting ORC DNA binding (68). In a previous work, we have analyzed nucleosome positioning (where nucleosomes are located at genomic DNA sequence) and occupancy (nucleosome density in a cell population) in the CL Brener genome by MNase-seq, a widely used methodology to map nucleosome positioning and occupancy (MNase) to digest DNA not bound to nucleosomes, leaving DNA-binding mononucleosomes that were purified and then sequenced (55). We then analyzed whether there is any correlation between Orc1Cdc6 peaks and nucleosome positioning in *T. cruzi*, an organism whose chromatin structure can be different from other eukaryotes since chromosome condensation is not observed during mitosis (69), and its histone primary sequence is variable (70). To perform the analysis, we obtained Orc1Cdc6 peaks that composed the Predominant, Flexible, or Dormant origin data sets and submitted them to a heatmap followed by hierarchical clustering against nucleosome position (Fig. 6A and B). We could see Orc1Cdc6 from the three different classes of origins within nucleosomes. In addition, origins are not randomly distributed through nucleosome neighborhoods, but they are grouped into different clusters: one where Orc1Cdc6 is upstream of nucleosomes (see, e.g., cluster 2 of Dormant in Fig. 6A) and one where origins are downstream of nucleosomes (see, e.g., cluster 3 of Dormant in Fig. 6A). Also, we found a third cluster where Orc1Cdc6 is within nucleosomes (see, e.g., cluster 1 at Predominant heatmap in Fig. 6A). We did not find any correlation between the position of Orc1Cdc6 in relation to nucleosomes and the transcription orientation of the locus. Moreover, there is a concentration of Orc1Cdc6 in Flexible origins upstream of nucleosomes.

Previous work containing an analysis of the nucleosome landscape in the CL Brener genome identified dynamic nucleosomes, which are different among *T. cruzi* life forms. Dynamic nucleosomes are enriched at multigenic families at dSSRs and can be classified into different categories, one of which is called “occupancy” nucleosomes, which are those whose occupancy level between life forms changes for a given genomic location (55). Therefore, we further searched if this specific class of dynamic nucleosomes could be differentially related to Orc1Cdc6 peaks corresponding to Predominant, Flexible, or Dormant origins (Fig. 6C and D). In fact, Orc1Cdc6 peaks from Dormant origins are within and flanking “occupancy nucleosomes,” while Orc1Cdc6 peaks from Predominant and Flexible overlap the nucleosomes, albeit fewer. These results raise the possibility of an epigenetic regulation of origin activation in *T. cruzi* that should be further investigated.

## DISCUSSION

In this work, we provided the location of replication origin in the *T. cruzi* CL Brener genome. Moreover, we presented the distribution of Predominant, Flexible, Dormant, and free origins of Orc1Cdc6 in different genome compartments and features. With these data, we created an atlas of origins in *T. cruzi* (Tables 3 and 4).

**TABLE 3:** Summary of enrichment of DNA replication origins in *T. cruzi* CL Brener Esmeraldo-like genome features

Origin type	Genome features where origins are enriched
Predominant	–^*b*^
Flexible	snoRNA
Dormant	snoRNA
	Inter-PTU
Orc1Cdc6 free	n.a.^*a*^

^*a*^ Not analyzed.

^*b*^ The dash represents that there was no statistically significant difference.

**TABLE 4:** Summary of enrichment of DNA replication origins in *T. cruzi* CL Brener Esmeraldo-like genome compartments and multigenic families

Compartment where origins are enriched		Genes where origins are enriched
Predominant	Both	DGF-1
Flexible	Both	DGF-1
		Retrotransposon
	Disruptive	Mucin
		MASP
		TS
Dormant	Both	DGF-1
		Retrotransposon
	Disruptive	Mucin
		MASP
		TS
Orc1Cdc6 free	Core	n.a.^*a*^

^*a*^ Not analyzed.

We first searched Orc1Cdc6-binding sites to understand the genomic location where origins were licensed. Although we found enrichment of Orc1Cdc6 in some regions of the genome, no consensus sequence was detected (data not shown). The enrichment of Orc1Cdc6 in inter-PTU is different from what was observed in mammals or even in *T. brucei*. In the first case, origins are in transcription start sites to avoid frontal conflicts between transcription and replication machinery (22). In the second case, in which *T. brucei* contains genes organized in polycistrons, Orc1Cdc6-binding sites were found in the boundaries of gene clusters, particularly in dSSRs (27). The presence of putative origins at dSSRs observed in *T. brucei* avoids frontal clashes between transcription and replication machinery, while the presence in inter-PTU observed in *T. cruzi* makes one fork go in the same direction of transcription but the other one goes in the opposite direction, clashing with transcription machinery. Interestingly, however, Predominant and Flexible origins are not fired in inter-PTU, meaning that these sites work as Dormant origins.

We found that Orc1Cdc6-binding sites were enriched in the Disruptive compartment and in DGF-1, GP63, and RHS (present in the Both compartment). This shows a preferential interaction of Orc1Cdc6 with specific genomic regions and demonstrates that different from what is observed in other organisms, in *T. cruzi*, Orc1Cdc6 is located within genes. In addition, when we compared the ChIP-seq data containing Orc1Cdc6-binding sites with fired origins from MFA-seq (what we called Predominant origins), we found that indeed initiation of replication is enriched in DGF-1 genes. MFA-seq analysis had already shown that origins fired frequently in a population are enriched in DGF-1 genes (32); thus, the matching of MFA-seq with ChIP-seq confirms that indeed MFA-seq origins (at least part of them) are those activated by Orc1Cdc6. Why the Predominant origins are fired at DGF-1 remains to be understood. We proposed that it favors replicative stress (by collision with transcription machinery) at DGF-1, increasing genetic variability at these genes (32), since DNA replication stress can give rise to mutations, gene number variation, and chromosome copies (12, 13), phenomena observed in *T. cruzi* (71). However, this hypothesis only makes sense if DGF-1 is transcribed. It has already been proposed that since DGF-1 genes are surrounded by pseudogenes, preserved DGF-1 might be under selective pressure and therefore might be transcribed (8). In addition, we have shown that origins containing DGF-1 present a higher number of SNPs when compared with DGF-1 that do not contain origins (32). Alternatively, the presence of Orc1Cdc6 at DGF-1 causing head collision with transcription machinery may be simply because these genes are nonessential. Further studies investigating the role of DGF-1 in *T. cruzi* will shed light on this discussion. We have also compared the ChIP-seq data containing Orc1Cdc6-binding sites (ChIP-seq data) with fired origins from D-NAscent (excluding MFA-seq origins) (what we called Flexible origins). From this data set, we found that Flexible origins that fire in a locus licensed by Orc1Cdc6 are within multigenic families DGF-1, MASP, Mucin, Retrotransposon, and TS. In the end, the scenario is that Predominant origins are located in DGF-1 genes, and Flexible origins are located in the remaining multigenic families, including DGF-1. Again, why does *T. cruzi* rely on replication initiation within virulence factor genes? The highly plastic multigenic family encoding fast-evolving surface antigens is enriched with origins, and therefore, the same explanation used above can be cited here: to generate genetic variability by correcting DNA damage caused by transcription-replication conflicts in these elements whose diversity is important for infection capacity in different cells and for escape from the immune system. In this direction, the presence of origins in the Disruptive compartment and in DGF-1, GP63, and Retrotransposon could contribute to the different evolution of these regions. In fact, chromosomal break ends, which could be generated by transcription-replication conflicts, are present in surface multigenic family genes (such as TS and mucins), and MASP genes are affected by genomic rearrangements in *T. cruzi* TcI isolates (10), showing that these multigenic families are subject to recombination. The involvement of origins in these events, although very suggestive, remains to be proven. The involvement of origin with genetic events is not a new idea in *Trypanosoma* since it has already been proposed that origin activation might be involved in VSG switching in *T. brucei*, where transcriptionally active VSG loci are replicated in early S, while silent VSG loci are replicated in late S. In this scenario, the conflict between transcription and replication machinery would trigger DNA damage that would drive VSG recombination (28, 63). An alternative to the hypothesis that the presence of Orc1Cdc6 in multigenic families favors genetic variability and/or genomic plasticity could be that Orc1Cdc6 in these loci impairs transcription of these genes that might be important in infective forms but not in replicative forms. If so, it would represent a level of transcriptional control in an organism that lacks promoters to turn off transcription in specific genes.

In addition, we found D-NAscent origins that did not overlap with Orc1Cdc6-binding sites. Notably, these origins are enriched in the Core compartment. Although it is possible that we failed to detect Orc1Cdc6 peaks in these regions, it is also possible that these origins fire independently of Orc1Cdc6 but, instead by MCMs, repositioned by transcription or by break-induced recombination; both processes have already been proposed in *Leishmania* (30, 31). In this scenario, we would have Orc1Cdc6-licensed origins at multigenic families and origins free of Orc1Cdc6, driven by transcription or repair, firing in the Core compartment. However, why does the presence of origins in the Core compartment not contribute to rapid genetic variability? The frequency of Orc1Cdc6-independent origins may be lower than the frequency of origins fired at Orc1Cdc6-binding sites. Studies are being conducted to test this hypothesis.

We described the presence of backup origins—those Orc1Cdc6-binding sites that did not match with fired origins (called Dormant). The presence of these Dormant origins can be very important in *T. cruzi* since this parasite might handle significant replicative stress, firing many more origins than the minimal necessary (72). These Dormant origins were also located in multigenic families (Tables 3 and 4). The same location observed for Dormant and Flexible origins allowed us to hypothesize that they belong to the same origin group, making two inferences:

- Multigenic families are binding sites for Orc1Cdc6. Human ORC1 may recognize origins through the G4 structure (73); thus, further studies are necessary to search for the presence of this structure in the *T. cruzi* genome and to detect whether Orc1Cdc6 has an affinity for G4. Additionally, chromatin structure might be involved in this Orc1Cdc6 preference (see below).
- Flexible origins could be Dormant origins that were activated as a consequence of replicative stress. In fact, we have shown in a previous work that *T. brucei* activates backup origins as a consequence of replicative stress caused by transcription (74). Thus, it is possible that in *T. cruzi*, which also presents constitutive transcription of gene clusters, the same process occurs.

In fact, Orc1Cdc6-licensed origins located at inter-PTUs, where no transcription occurs, are not activated (Tables 3 and 4). In this direction, one possibility is that when replicative stress driven by transcription occurs in polycistronic CDS, closer backup origins still within genes are activated, preserving the activation of inter-PTU Dormant origins.

We created a data set of Predominant and Flexible origins, and therefore, we could compare the presence of Orc1Cdc6 within origins and at their boundaries. Interestingly, Predominant origins are fired closer to Orc1Cdc6 than Flexible ones. As cited before, the number of loaded MCMs can influence the probability of an origin being fired (67); therefore, the difference between distances may be a consequence of MCM organization and the formation of the Cdc45-MCM2-7-GINS preinitiation complex in the locus. Corroborating these data, the density of Orc1Cdc6 in origins correlates with the frequency of origin firing. Therefore, it is time to speculate if the presence of Orc1Cdc6 within DGF-1 genes, where Predominant origins accumulated, correlates with this phenomenon. The structure of chromatin in different regions of the genome may be involved in this process. In fact, it has already been shown that chromatin structure is related to transcriptional processes since the chromatin structure reflects gene expression (55); thus, it is reasonable that chromatin contributes to DNA replication regulation as well, as discussed below. The relationship between Orc1Cdc6 density and origin firing helps to understand the molecular bases of the firing of replication.

We found three patterns of nucleosome positioning in a 3-kb window of origin signals. In two of them (the majority), the origin is bound in a nucleosome-depleted region but surrounded by them. This is exactly what was observed in yeast, where origins are free of nucleosomes to allow protein-DNA binding (75). In addition, in metazoans, ORC interacts with open chromatin regions containing posttranslational modifications of specific histones (76). Therefore, it is tempting to speculate that nucleosome modifiers and remodelers are involved in the dynamics of DNA replication in *T. cruzi*. Finally, nucleosomes that present different occupancies between *T. cruzi* life forms are distinctly associated with the different origin classes. Orc1Cdc6 found at Predominant and Flexible origins tend to be located upstream these nucleosomes, while Dormant origins are within this class of nucleosomes. It suggests that occupancy nucleosomes can inhibit origin firing even when do not inhibiting ORC-DNA binding. Since these nucleosomes change during *T. cruzi* infective/nonreplicative and replicative/noninfective forms (55), it is possible that chromatin structure alteration is involved in the modulation of DNA replication during the *T. cruzi* life cycle. The involvement of chromatin in this modulation would be one more reason for the lack of replication in infective forms since we have already shown that infective forms lack MCM7, Orc1Cdc6-DNA interaction (77), and RPA in the nuclear space (78).

As cited, in *T. brucei,* origins were found at SSRs (27) and, in *Leishmania,* at SSRs or in no specific region (depending on the methodology used) (29). Here, we effectively contributed to show location of origins in *T. cruzi*, filling the scenario of origin location in trypanosomatids. Since these parasites present distinct life cycle and different tactics of infection, the location of origins considering the genome context might reflect different mechanisms to maintain and to evolve the genome. The molecular mechanisms involved in the genomic maintenance (with strategically variations) might have direct consequences in the pathobiology of the diseases these organisms propagate.

In summary, we dissected the location of different classes of origins in the *T. cruzi* genome and their relationship with local chromatin. These results allowed us to propose the following hypothesis: chromatin structure allows the interaction of Orc1Cdc6 with multigenic families. Dynamic nucleosomes, together with a higher Orc1Cdc6 density, promote the activation of origins at DGF-1, while replicative stress mostly driven by transcription activates origins in the remaining multigenic family genes, including DGF-1. Finally, Orc1Cdc6-independent origins, activated by transcription and repair, fire at the Core compartment. Understanding the molecular bases of DNA replication in *T. cruzi* and their involvement in genome evolution is important to understand how this parasite deals with the pressure of the host immune system. Moreover, detailed studies of replication initiation occur in a limited number of organisms. This work explored replication initiation in *T. cruzi*, which belongs to a different branch of the eukaryotic domain, providing insights into the diversity of this essential biological process.

## Data Availability

NCBI’s BioProject related to this paper is PRJNA1002335. The accession numbers for the ChIP-seq BioSample (and respective SRA IDs) of data reported in this paper are SAMN36840633 (SRR25628529), SAMN36840634 (SRR25628528), SAMN36840635 (SRR25628520), SAMN36840636 (SRR25628519), SAMN36840637 (SRR25628518), SAMN36840638 (SRR25628517), SAMN36840639 (SRR25628516), SAMN36840640 (SRR25628515), SAMN36840641 (SRR25628514), SAMN36840642 (SRR25628513), SAMN36840643 (SRR25628527), and SAMN36840644 (SRR25628526). The ones relative to D-NAscent samples are SAMN36840645 (SRR25628525), SAMN36840646 (SRR25628524), SAMN36840647 (SRR25628523), SAMN36840648 (SRR25628522), and SAMN36840649 (SRR25628521). The developed code used to run all the computational analyzes is available at the following GitHub page: https://github.com/davidsilvapires/tcruziReplicationOrigins.git.

## SUPPLEMENTAL MATERIAL

Fig. S1 to S12 mbio.00319-24-s0001.pdf Workflows, characterization of lineage, and additional large-scale analysis.

ASM does not own the copyrights to Supplemental Material that may be linked to, or accessed through, an article. The authors have granted ASM a non-exclusive, world-wide license to publish the Supplemental Material files. Please contact the corresponding author directly for reuse.
