# Supplementary material for: Integrating high-throughput analysis to create an atlas of replication origins in Trypanosoma cruzi in the context of genome structure and variability
Source: mBio. 2024 Mar 5;15(4):e00319-24. doi: 10.1128/mbio.00319-24 (PMC11005370; doi:10.1128/mbio.00319-24)
Supplement: Fig. S1 to S12 — Workflows, characterization of lineage, and additional large-scale analysis. [file mbio.00319-24-s0001.pdf]

Integrating high-throughput analysis to create an atlas of replication origins in *Trypanosoma cruzi* in the context of genome structure and variability

Supplemental material

Marcela de Oliveira Vitarelli, Thiago Andrade Franco,
David da Silva Pires, Alex Ranieri Jerônimo Lima,
Vincent Louis Viala, Amelie Johanna Kraus,
Inácio de Loiola Meirelles Junqueira de Azevedo,
Julia Pinheiro Chagas da Cunha, Maria Carolina Elias

List of Supplementary Figures

1 Predominant origins pipeline 2
2 Flexible origins pipeline 2
3 Dormant origins pipeline 3
4 Orc1Cdc6-free pipeline 3
5 Genome Compartment Pipeline 4
6 Construction of the Orc1Cdc6 lineage fused to the 3xTy1 tag . . 5
7 Analysis between Orc1Cdc6 peaks and correlation with *T. cruzi* chromosomes 6
8 Distribution of ChIP-seq Orc1Cdc6 peaks along the chromosomes 7
9 RawData of heatmap and hierarchical cluster of Genome-wide localization of Orc1Cdc6 peaks 8
10 Hierarchical clusters Orc1Cdc6 peaks mean concerning MFA-seq origins 9
11 MEME analysis 10
12 Orc1Cdc6-free analysis 11

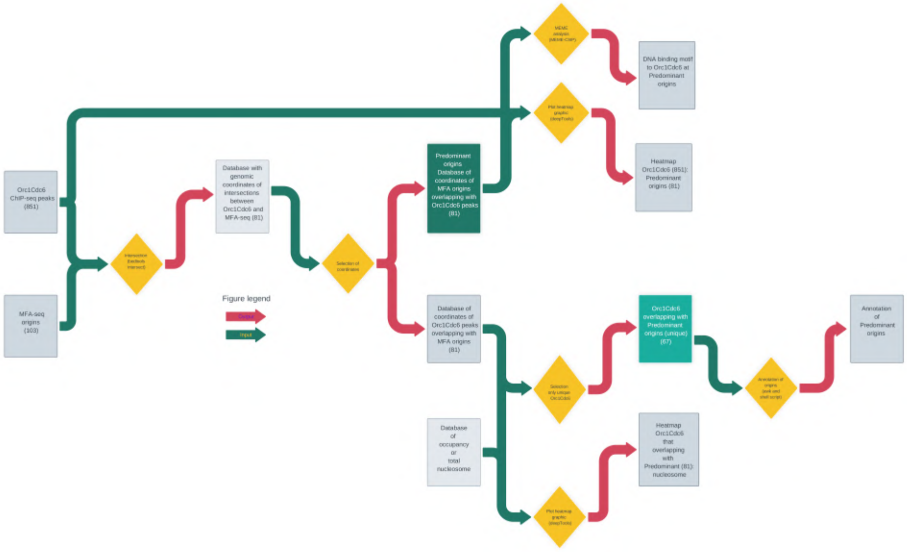

Supplementary Figure 1: **Predominant origins pipeline.** Flowchart of setup and processing of the Predominant origins database.

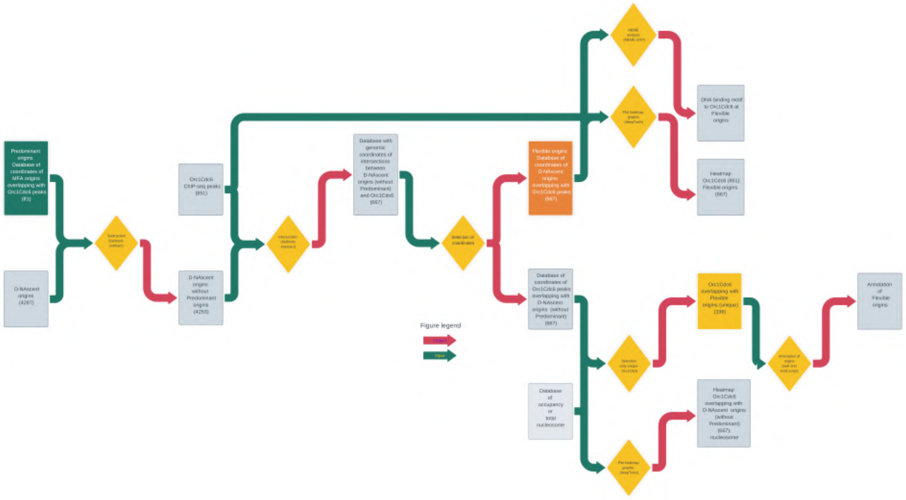

Supplementary Figure 2: **Flexible origins pipeline.** Flowchart of setup and processing of the Flexible origins database.

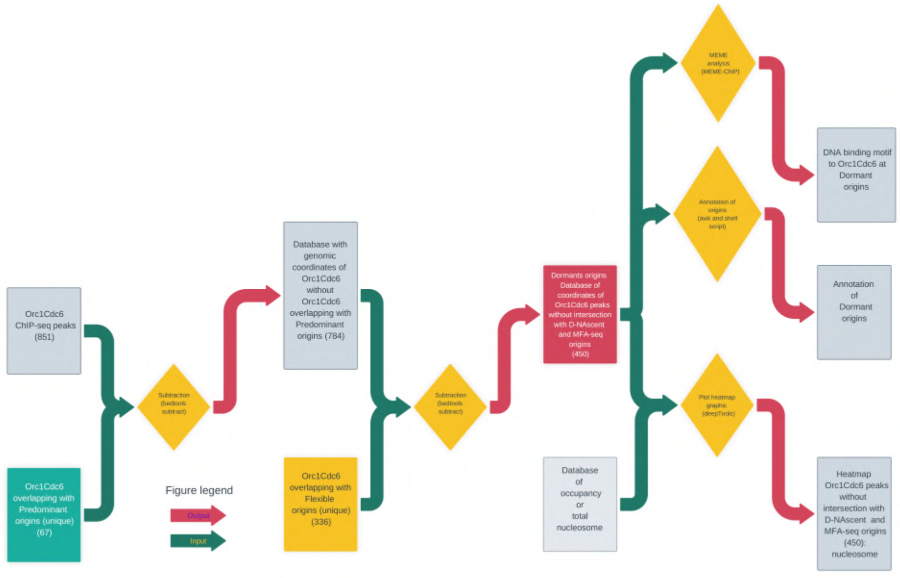

Supplementary Figure 3: **Dormant origins pipeline.** Flowchart of setup and processing of the Dormant origins database.

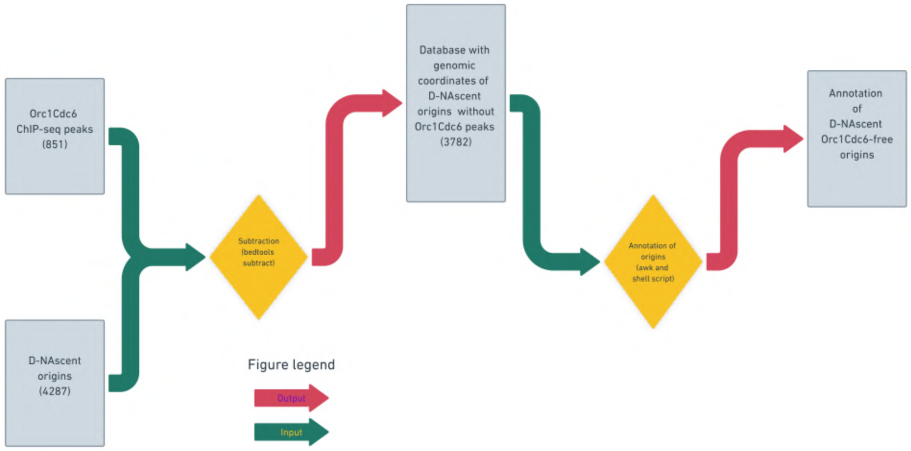

Supplementary Figure 4: **Orc1Cdc6-free pipeline.** Flowchart of setup and processing of the Orc1Cdc6-free origins database.

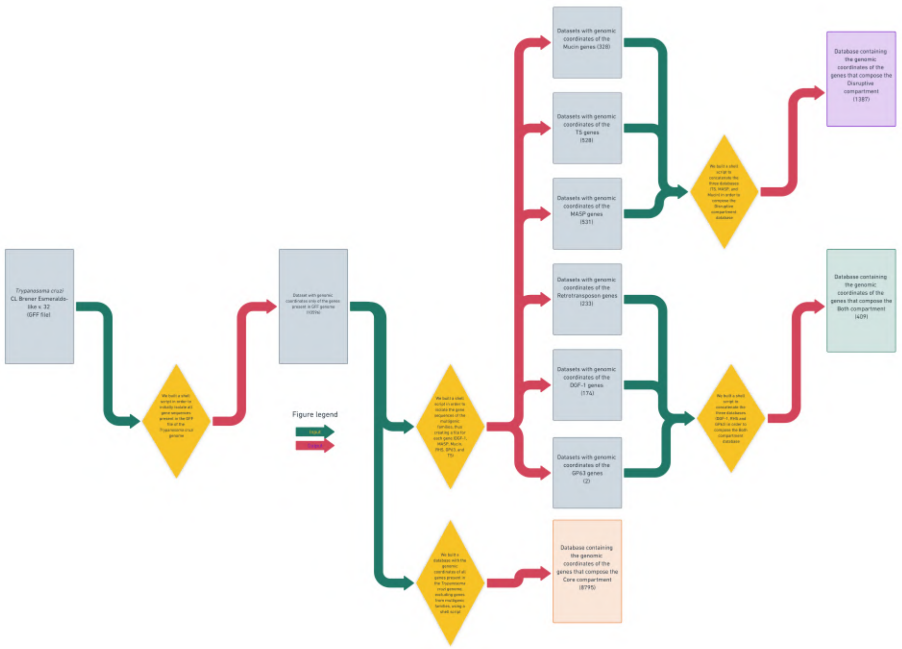

Supplementary Figure 5: **Genome Compartment Pipeline.** Flowchart of setup and processing of the Genome Compartment of *T. cruzi* database.

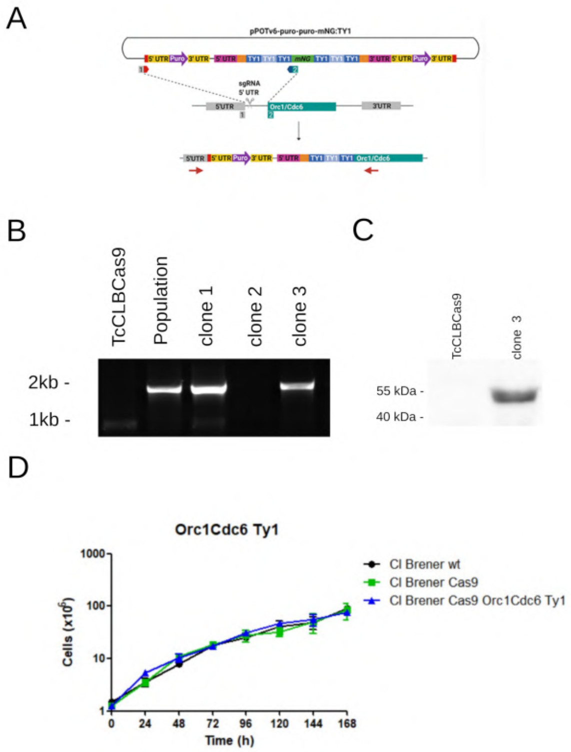

Supplementary Figure 6: **Construction of the Orc1Cdc6 lineage fused to the 3xTy1 tag.**

(A) Representative scheme of the pPOTv6-puro-puro-mNG:TY1 plasmid containing the puromycin resistance gene (puro) and 3xTy1 tag. Numbers 1 and 2 represent the 30 bp anchor regions used for amplification and insertion of the 3xTy1 tag at the N-terminal region of TcOrc1Cdc6. Red arrows indicate the location of primers designed to confirm tag positioning. Image created with BioRender.com.

(B) PCR detection of TcOrc1Cdc6-tag using the primers represented by red arrows in (A). The agarose gel shows the result of PCRs performed with different DNA templates: *T. cruzi* CL Brener Cas9 as a negative control, CRISPR–Cas9 transfected population and three different clones. The approximately 2 kb PCR product corresponds to the expected size of the TcOrc1Cdc6-tag fragment (1,751 bp).

(C) Western blot analysis of control and TcOrc1Cdc6-Ty1 clone 3 with anti-Ty1 tag antibody.

(D) Growth curves with *T. cruzi* CL Brener wild type, Cas 9 and Orc1Cdc6-Ty1 clone 3 strains were performed in triplicate starting from a 1×10^6 cells/mL dilution. The bars represent the standard deviation.

A

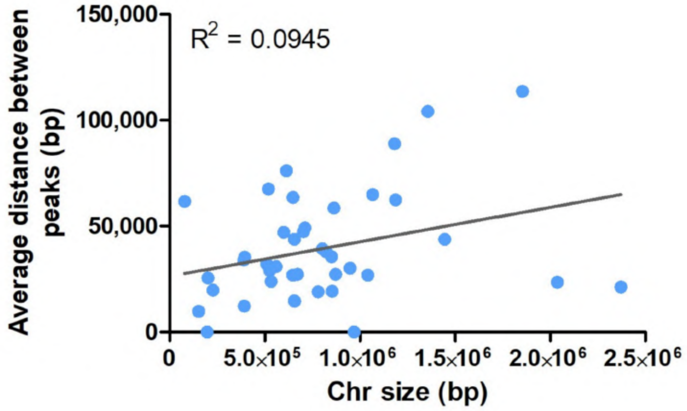

B

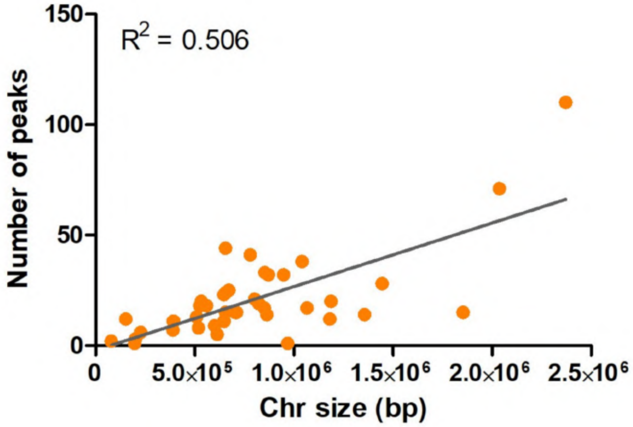

Supplementary Figure 7: **Analysis between Orc1Cdc6 peaks and correlation with *T. cruzi* chromosomes.**
(A) Linear regression between the distance of Orc1Cdc6 peaks and the size of the chromosomes.
(B) Linear regression between the number of Orc1Cdc6 peaks and chromosome size. R2 values are shown on the top right of each graph along with the trend lines for Orc1Cdc6 peaks.

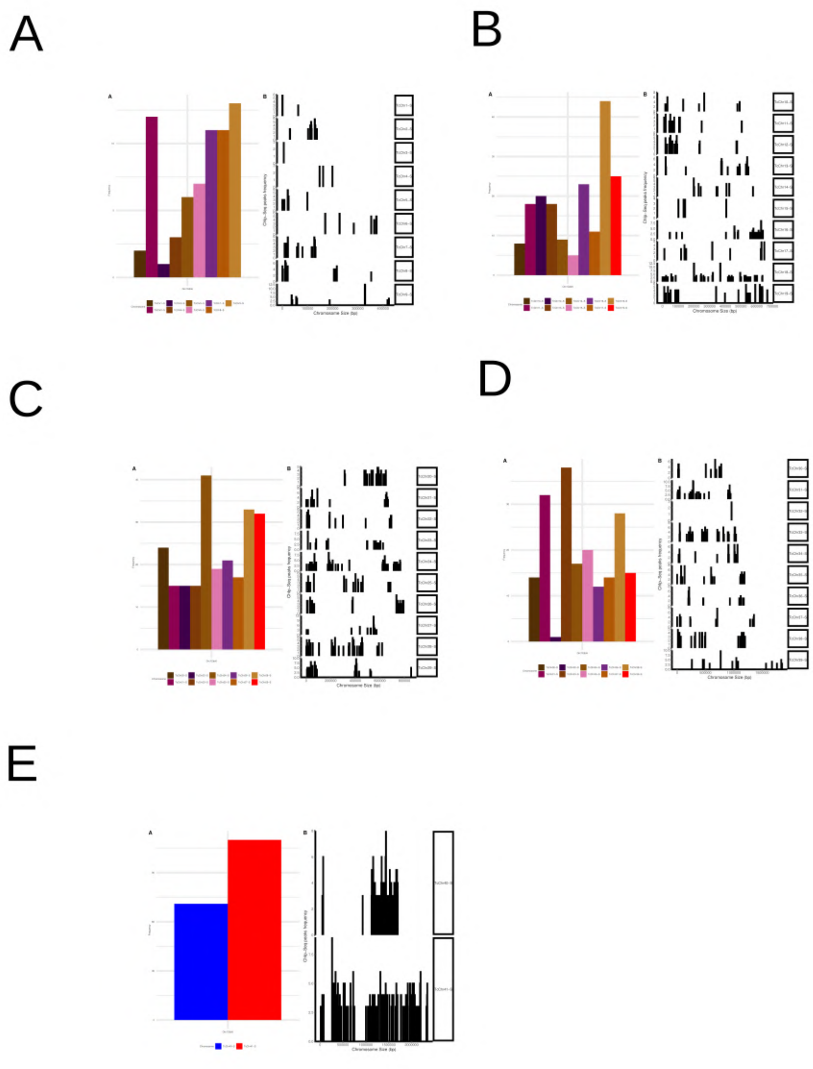

Supplementary Figure 8: **Distribution of ChIP-seq Orc1Cdc6 peaks along the chromosomes.**
(A) Orc1Cdc6 over chromosomes 1 to 9.
(B) Orc1Cdc6 over chromosomes 10 to 19.
(C) Orc1Cdc6 over chromosomes 20 to 29.
(D) Orc1Cdc6 over chromosomes 30 to 39.
(E) Orc1Cdc6 over chromosomes 40 and 41.

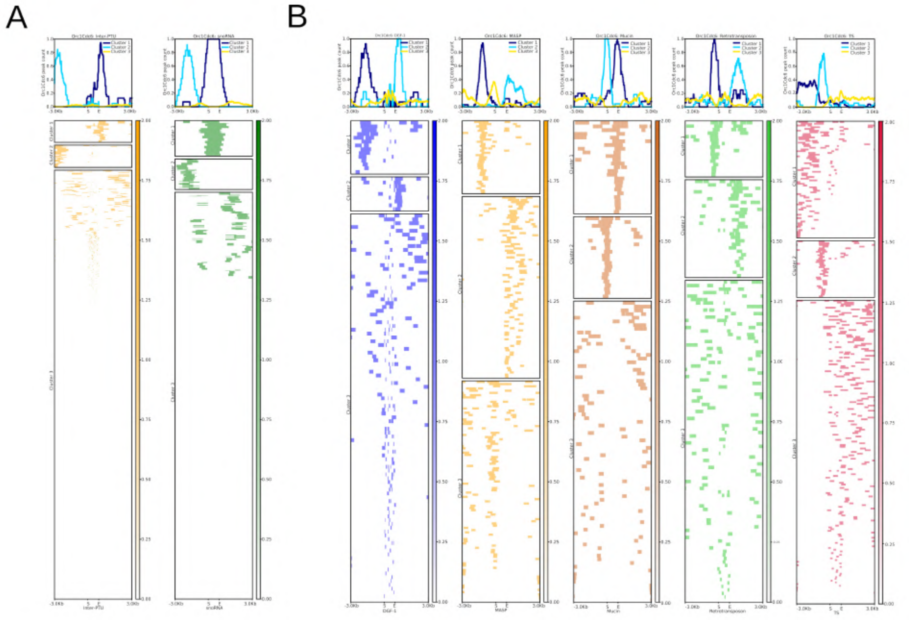

Supplementary Figure 9: **RawData of heatmap and hierarchical cluster of Genome-wide localization of Orc1Cdc6 peaks.**

(A) Heatmaps for Orc1Cdc6 peaks mean concerning inter-PTU and snoRNA region considering a ±3 kb window.

(B) Heatmaps for Orc1Cdc6 peaks mean concerning genes for multigenic family proteins (DGF-1, MASP, Mucin, Retrotransposon and TS) considering a ±3 kb window.

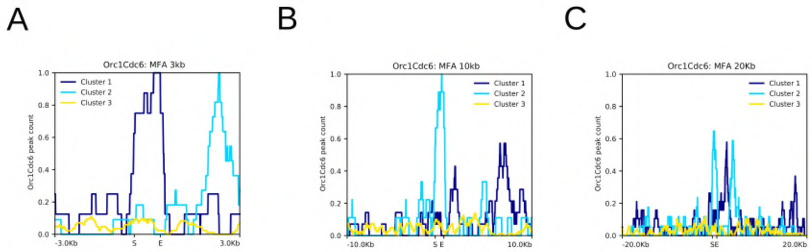

Supplementary Figure 10: **Hierarchical clusters for Orc1Cdc6 peaks mean concerning MFA-seq origins.**
(A) ±3 kb window.
(B) ±10 kb window.
(C) ±20 kb window.

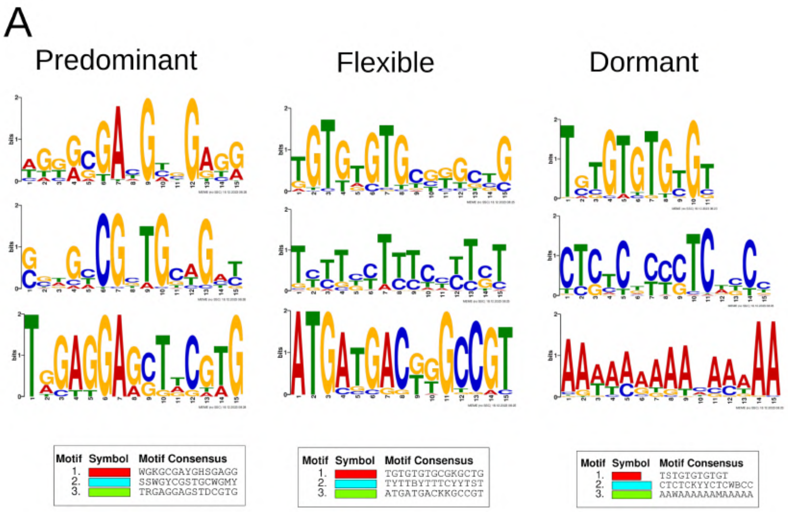

B

Predominant

Motif Found	E-value	Known or Similar Motifs
WGKGCGAYGHSGAGG	8.9e-011	Not found in this database
SSWGYCGSTGCWGMY	5.7e-010	Not found in this database
TRGAGGAGSTDCGTG	4.3e-003	Not found in this database

Flexible

Motif Found	E-value	Known or Similar Motifs
TGTGTGTGCGKGCTG	1.6e-111	Not found in this database
TYTTBYTTTCYYTST	1.4e-067	Azf1p (Azf1p&consensus=aAAAAGAAa) - Transciption Factor - Badis et al., 2008
ATGATGACKKGCCGT	1.8e-059	Not found in this database

Dormant

Motif Found	E-value	Known or Similar Motifs
TSTGTGTGTGT	2.5e-096	Rap1p (Rap1p&consensus=ACACCCAYACAYYY) and Rap1p (Rap1p&consensus=ACACCCRYACAY) Transcriptor Fator - Idrissi & Piña, 1999 ; Lieb et al., 2001
CTCTCKYYCTCWBCC	1.6e-049	Haa1p (Haa1p&consensus=GGCGAGAGG) – Transcriptor Factor – Mira et al., 2011
AAWAAAAAAMAAAAA	6.7e-042	Azf1p (Azf1p&consensus=aAAAAGAAa) Hcm1p (Hcm1p&consensus=WAAYAAACAAW) Azf1p (Azf1p&consensus=AAAAGAAA) – Transcripto Facto - Badis et al., 2008; Zhu & Davis, 1998; ;Slattery et al., 2006; Laura et al., 2002

Supplementary Figure 11: **MEME analysis.** DNA binding motifs for the Predominant, Flexible, and Dormant replication origins identified through analyses using the MEME-ChIP software.

(A) Representative logos of DNA binding motifs with the highest e-value identified among the genomic coordinates of Predominant, Flexible, and Dormant DNA replication origins found by MEME-ChIP.

(B) Summarized table of the top 3 motifs with the highest e-values.

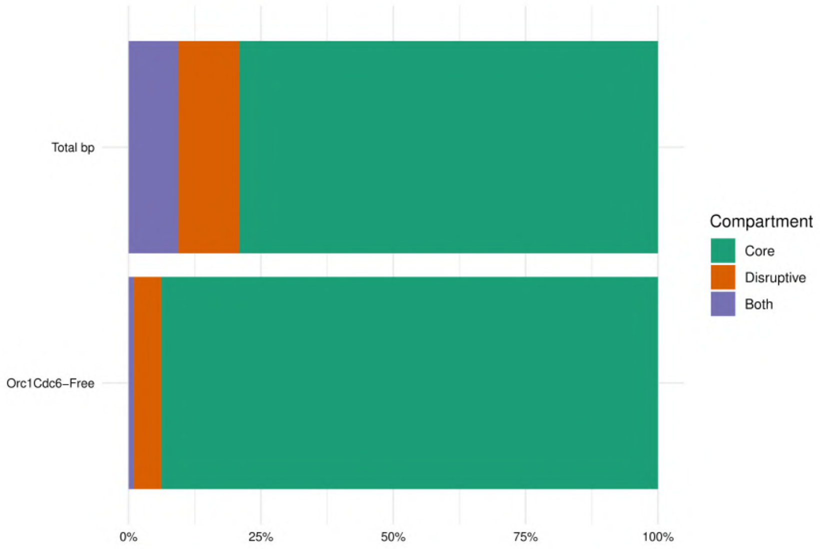

Supplementary Figure 12: **Orc1Cdc6-free analysis.** Bar graph depicting DNA replication origins from D-NAscent subtracted from Orc1Cdc6 peaks distributed across genome compartments. Statistical significance tests were performed with the chi-square goodness of fit test using the standard frequency 83.04% Core, 13.10% Disruptive, and 3.86% Both, and post hoc test Cramer's V. The values $p = 2.2 \times 10^{-16}$, ${X^2}_{(2)} = 979.96$, V-Cramer = 0.05772414 were obtained.
